# Single Base-Resolution Methylome of the Dizygotic Sheep

**DOI:** 10.1371/journal.pone.0142034

**Published:** 2015-11-04

**Authors:** Yangzi Wang, Jianghong Wu, Xiao Ma, Bin Liu, Rui Su, Yu Jiang, Wen Wang, Yang Dong

**Affiliations:** 1 Kunming University of Science and Technology, Chenggong District, Kunming, China; 2 State Key Laboratory of Genetic Resources and Evolution, Kunming Institute of Zoology, Chinese Academy of Sciences, Kunming, China; 3 Animal Husbandry Institute, Inner Mongolia Academy of Agricultural & Animal Husbandry Sciences, Hohhot, China; 4 Inner Mongolia Prataculture Research Center, Chinese Academy of Science, Hohhot, China; 5 Yunnan Agricultural University, Kunming, China; 6 College of Animal Science, Inner Mongolia Agricultural University, Hohhot, China; 7 College of Animal Science and Technology, Northwest A&F University, Yangling, China; Harbin Medical University, CHINA

## Abstract

Sheep is an important livestock in the world for meat, dairy and wool production. The third version of sheep reference genome has been recently assembled, but sheep DNA methylome has not been profiled yet. In this study, we report the comprehensive sheep methylome with 94.38% cytosine coverage at single base resolution by sequencing DNA samples from *Longissimus dorsi* of dizygotic Sunit sheep, which were bred in different habitats. We also compared methylomes between the twin sheep. DNA methylation status at genome-scale differentially methylated regions (DMRs), functional genomic regions and 248 DMR-containing genes were identified between the twin sheep. Gene ontology (GO) and KEGG annotations of these genes were performed to discover computationally predicted function. Lipid metabolism, sexual maturity and tumor-associated categories were observed to significantly enrich DMR-containing genes. These findings could be used to illustrate the relationship between phenotypic variations and gene methylation patterns.

## Introduction

DNA methylation at the 5 position of cytosine is common in genomes of fungi, plants and animals [1, 2]. This epigenetic phenomenon is known to silence exogenous transposons, imprint genes and regulate gene expression [3]. In animals, DNA methylation has been implicated in memory formation [4, 5] and carcinogenesis [6–9], which demonstrates its critical contributions to development, physiology and phenotypic variation [10].

Studies in *Arabidopsis* and zebrafish showed that DNA methylation patterns could be inheritable from parents to offspring [11, 12]. However, the methylation patterns may be different between offspring and their parents, possibly due to the demethylation process in gametes before fertilization and the methylation reestablishment after fertilization [13–15]. This mechanism may produce divergent phenotypes capable of responding to changing environment through regulation of gene expression [16].

Sheep (*Ovis aries*) is one of the first domesticated mammals for meat, dairy and wool production. Genomic studies on this important livestock have been trying to facilitate the raising and breeding process for desirable traits. So far, sheep reference genome has been assembled [17], and comprehensive maps of functional elements of sheep are also being profiled [18]. However, limited sheep DNA methylation analysis at specific sequences was reported [19]. Therefore, single base resolution DNA methylome with whole genome coverage in sheep is warranted for the investigation of complex epigenetic regulation.

Whole genome bisulfite sequencing (BS-seq) was first used in *Arabidopsis thaliana* DNA methylome studies [20, 21], and was proved to be effective to profile accurate, unbiased and high-coverage DNA methylation landscapes in model animals like human, mice and rat [3, 22, 23]. However, such powerful tool could hardly be seen in livestock DNA methylation studies. Here, we described the comprehensive sheep *Longissimus dorsi (LD)* muscle DNA methylome with 94.38% cytosine coverage at single base resolution using BS-seq. This high coverage sheep *LD* methylome provides resource to profile widespread genomic CG and non-CG methylation (CHG and CHH contexts (H = A, T or C)) patterns. This resource not only provides insight into the development of animal husbandry, but also helps understand the process of species evolution and differentiation during domestication.

## Materials and Methods

### Ethics statement

The feeding and the care of lambs, the euthanasia and other research process were strictly performed in accordance with international guidelines and ethical standards. The protocol of this research has been reviewed and approved by the Ethics and Experimental Animal Committee of Kunming Institute of Zoology, Chinese Academy of Science.

### DNA samples preparation and BS-seq sequencing

Dizygotic 6-month-old female twin sheep were chosen as DNA sample donors. These twin sheep were parted immediately after birth and raised in different environments. One free-range sheep grazed with a flock in a desert grassland environment, which enjoyed natural pasture and is defined as Sheep A. The twin sister (Sheep B) was house-raised; ate corn, cornstalk and wheat bran. Both sheep were euthanized (performed by skilled veterinarian via intravenous injection of sodium pentobarbital (100mg/kg)) at the age of six months for muscle samples from *Longissimus dorsi*. After DNA sample extracted, a bioruptor (Covaris S220) was used to fragment sheep genomic DNA to a mean size of approximately 500 bp. After fragmentation, DNA fragments were blunt-ended, added dA at the 3’-ends, and then added adapters. The procedure was carried out in strict accordance with the Illumina manufacturer instructions. Adapters-added DNA were given a bisulfite conversion step, described in a previous study [24]. Bisulfite-treated DNA was PCR amplified and then pair-end sequenced with 90 read length of each end using Illumina high throughput sequencing system (Hiseq 2500).

### BS-seq reads mapping and methyl-cytosines analysis

Reads yielded from BS-seq were mapped to the well assembled sheep reference genome (Version 3.1) from Livestock Genomics (http://www.livestockgenomics.csiro.au/sheep/oarv3/Oarv3.1.alldna.lowercase.masked.fasta.gz) using Bismark (v0.10.0) [25]. Only one mismatch is permitted in the “seed” (the high quality end of the read, default is 28 bases) while aligning. Other parameters were set as default. The supplementary script “bismark_methylation_extractor” packaged in Bismark was used to extract methylation calls. Every reported cytosine site, with four or more covered reads was used to produce cytosine report. Further studies were all based on this report.

In addition, all reads were also mapped to the sheep mitochondrial genome (from NCBI, Accession NC_001941) to calculate the sum of non-conversion rate (non-conversion cases most produced in bisulfite treating process, others are T-C sequencing errors and single nucleotide polymorphisms (SNPs)).

### Identification of DMR-containing Genes

swDMR [26], a software based on sliding window approach, was used to identify DMRs. 100 bp steps, 1kb sliding windows [3] were used to go throughout two sheep *LD* methylomes. Only windows containing at least 10 CG sites were kept to calculate the mean methylation level. Between two samples, those windows have at least 2-fold and 0.1 mean methylation level differences were used to perform T-test (P < 0.01, FDR < 0.05) to identify differentially methylated windows. After that, each two of these differentially methylated windows were considered as one if their distance was less than 100 bp (include overlaps) to demarcation DMRs. Other parameters were set as default. Gene annotation of sheep reference genome v3.1 was downloaded from Livestock Genomics (http://www.livestockgenomics.csiro.au/sheep/oarv3/Oarv3.1.protein.gene.gff3), and used to annotate DMR-containing genes.

### GO and KEGG analyses of DMR-containing genes

Gene ontology analysis of DMR-containing genes was performed using InterProScan 5 [27]. DNA sequences of sheep annotated genes were computationally converted into polypeptide sequences, and then aligned to InterProScan protein database. GO terms with P value less than 0.05 were considered to be statistically significant. Enrichment analysis for DMR-containing genes in each region was performed using Ontologizer 2.0 [28] with their GO terms. All GO annotated sheep genes were listed as a background list. GO core annotation files (go.obo and go.owl) were downloaded from http://geneontology.org/page/download-ontology. Ontologizer was run using the Fisher’s exact test with P value < 0.05. KOBAS 2.0 is available at: http://kobas.cbi.pku.edu.cn/home.do. All options were default.

## Results

### Sheep DNA methylation whole-genome bisulfite sequencing and quality control

To study the general methylation pattern of sheep as well as the DNA methylation divergence induced by different habitat, we chose a pair of 6-month-old female twin sheep as DNA sample donors. These twin sheep were intentionally defined as sheep A and sheep B according to the different growing environments, and had large phenotype variant in weight at the age 6 months. Sheep B weighed up to 38.7 kg, about 15.2 kg heavier than Sheep A (23.5 kg). For these twin sheep, DNA sample was extracted from *Longissimus dorsi* of each sheep to generate whole-genome size DNA methylome maps using BS-seq method [29]. 786 million BS-seq raw reads were generated from Sheep A, and 805 million from Sheep B. Quality control process was performed to remove low quality reads (including those within Ns >10%, low quality sites > 40%, adaptor contamination, and duplication pairs). 452 million clean reads were obtained for Sheep A and 407 million for Sheep B. These reads were subsequently mapped to the *Ovis aries* reference genome (Oar v3.1) [17] using methyl-data alignment software Bismark [25].

### Sheep DNA methylome landscape profiling

Clean reads from the twin sheep were first mapped to the reference sheep genome separately. 268 million clean reads were uniquely mapped for producing Sheep A methylome and 234 million clean reads for Sheep B methylome. These two methylomes were stored and prepared for further comparison. By pooling together clean reads from both sheep, a total of 502 million clean reads were uniquely mapped to cover 94.38% cytosine of the reference genome with average 17.2 × high depths per strand (Table 1). This mapping result reached anticipated and desired completeness of the sheep *LD* methylome. In accordance with a recent study that reported mitochondrial genome in vertebrates lacking methylation [30], sheep mitochondrial genome from NCBI (NC_001941) was used to calculate the sum of non-conversion rate (non-conversion cases/total cases) at 0.33%. Non-conversion cases are mostly produced in bisulfite treating process, and others caused by T-C sequencing error and SNPs.

**Table 1:** Data description of BS-Seq reads for the twin sheep.

	Sheep A	Sheep B	Merged data
**Raw reads number**	785,849,911	804,900,159	1,590,750,070
**Raw data production (Gb)**	141.45	144.88	286.33
**Cleaned and uniquely mapped reads number**	268,396,277	233,607,387	502,003,664
**Clean data production (Gb)**	48.31	42.05	90.36
**Average reads depth per strand**	9.2×	8.0×	17.2×
**Reference genome/size (Gb)**	*Ovis aries* (v3.1) / 2.62		

All 96.8 million mC constituted about 9.79% of all cytosine in the sheep reference genome. About 45.3 million mC occurred in CG dinucleotide, 14.7million in CHG context, and 36.9 million in CHH context. They respectively accounted for 92.01% of total CG sites, 5.13% of total CHG sites and 6.64% of total CHH sites (Table 2).

**Table 2:** DNA methylation patterns in three contexts.

Cytosine contexts	CG	CHG	CHH	Total
**Methylated cytosine number**	45,260,666	14,661,439	36,864,132	96,786,237
**Methylation density (mC/C)**	92.01%	6.65%	5.13%	9.79%

**Fig 1:**
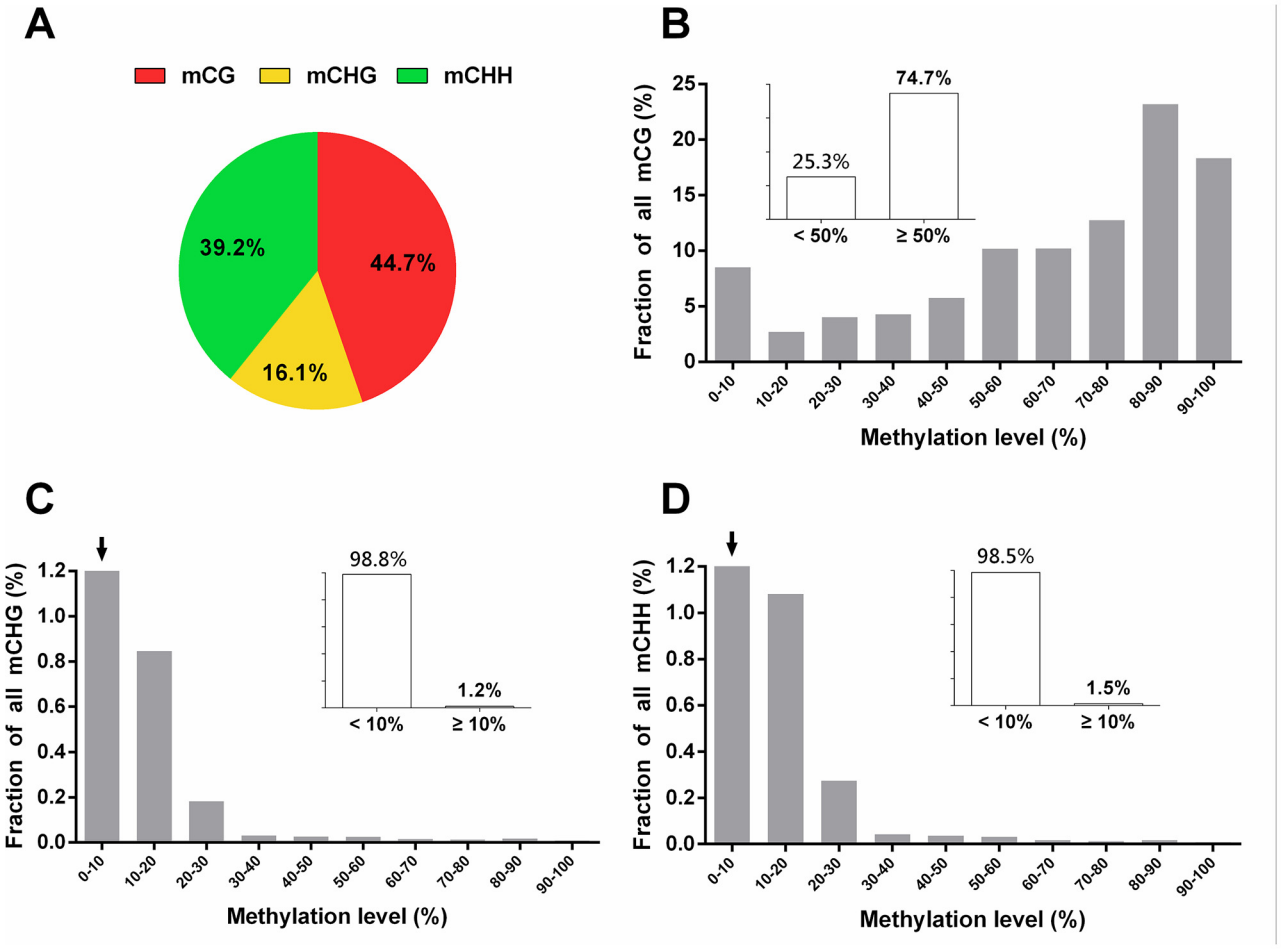
Sheep *LD* muscle DNA methylation patterns. (A) Three contexts of methylated cytosines relative proportions. (B) Distribution of CG methylated level. The y-axis demonstrates the proportion of mC level calculated within bins of 10%. The x-axis indicates methylation level at each reference cytosine, which defined as reads that support methylation divide depths. And only cytosine covered at least 4 reads were calculated. (C) Distribution of CHH methylated level, notice that the first bar with a black arrow symbol is not completely shown, the value of this bar is 98.48%. (D) Distribution of CHG methylated level. The value of the first bar with a black arrow is 98.84%.

The proportion of CG methylation is comparable to that of previously reported mouse and human methylomes [3, 23]. Strikingly, substantial proportion of non-CG methylation was observed in sheep *LD* methylome (Fig 1A). CG methylation occupied 44.73% of all methylated cytosine, while CHG and CHH contexts occupied 16.07% and 39.19% respectively. This result, together with previous findings of non-CG methylation in the mouse frontal cortex [23], human embryo stem cells [3] and several mammalian cell lines [31, 32], challenges the prevailing notion that non-CG methylation is rare or non-existent in mammals [33, 34].

The frequency of DNA methylation level of three contexts (CG, CHG and CHH) was profiled. Proportion of reads that supported methylation of covering depth at a specific site was generally defined as the methylcytosine (mC) methylation level. In this study, CG methylation level showed a bimodal distribution. About 74.71% methylated CG with level higher than 50% and a low peak appeared at level lower than 10% (Fig 1B). Despite a substantial amount of non-CG methylation, the methylation levels were very low, with more than 98% cytosines (CHG 98.84% and CHH 98.48%) with methylation level lower than 10% both in CHG and CHH contexts (Fig 1C and 1D).

Compared with Couldrey’s work, our result showed higher CG methylation density (92% vs. 50%-55%) in sheep *LD* methylome [19]. A possible explanation was that their approach was only focus on CG-riched genomic regions (covered ~1% of sheep genome), which were known to be frequently hypomethylated in mammals. Our observation that CG methylation level showed bimodal distribution is in accordance with that observed in [19], well supported the hypothesis that each CG methylation site is classified as methylated or unmethylated [19].

### Multilayer global scale views of sheep *LD* methylome

Multilayer global scale maps of sheep *LD* methylome were profiled. Perspectives from genome scale, chromosome scale and base resolution provided comprehensive and deep understanding of sheep *LD* methylome.

#### Genome scale map of sheep *LD* methylome

**Fig 2:**
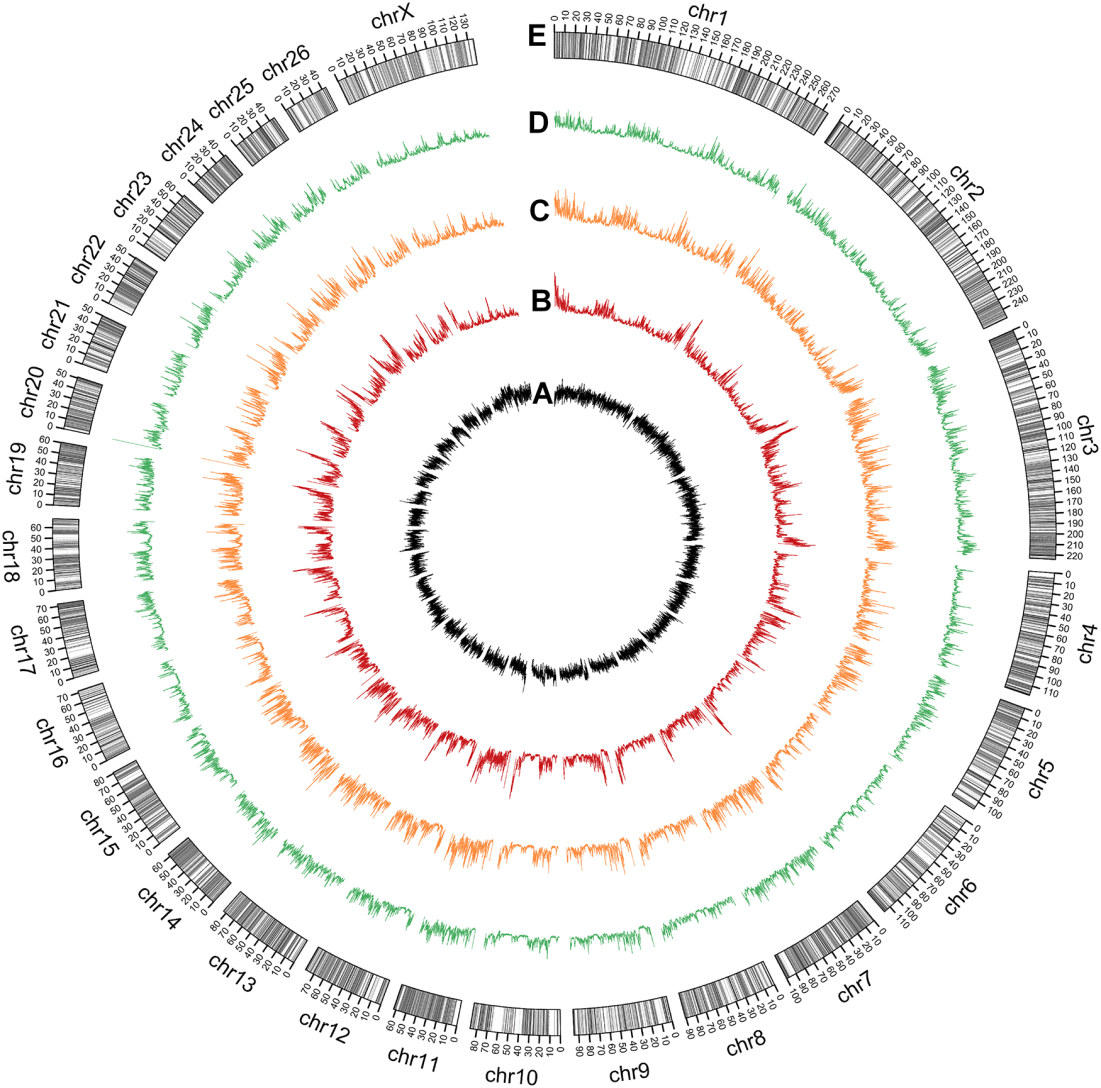
Whole-genome scale view of sheep *LD* muscle methylome. (A) Broken line chart of repeats density in 100 kb windows. (B) Red broken lines indicate normalized methylated cytosine density in CG context in 100 kb windows. (C) Orange broken lines indicate normalized mCHG density. (D) Green broken lines indicate normalized mCHH density. (E) Oar v3.1 chromosome bars. Highlighted gray bands refer to normal annotated genes.

Genome scale map of mC density (mC/C) and repeats density of sheep *LD* muscle were drawn with Circos [35]. We described repeats density distribution lines (Fig 2A), mC density of sheep *LD* methylome in three sequence contexts (CG, CHG and CHH) (Fig 2B, 2C and 2D), and gene locus (Fig 2E) were highlighted on each chromosome bar throughout the whole genome. Non-CG methylation density showed similar distribution with CG methylation. Especially at the edges of some chromosomes, all of three contexts methylation shared same rising trend. Interestingly, mC density in sheep *LD* methylome showed negative correspond distribution with repeats density in gene-rich districts and edges of chromosomes. At this genome scale layer, methylomes of twin sheep were also profiled separately (S1 Fig). As expected, mC density of two sheep in all three contexts appeared highly consistent, even at the differentially methylated regions (DMRs, highlighted as red on chromosome bars). This high consistency not only supported the reliability of the methylomes from twin sheep, but also confirmed the validity of the non-CG methylation in sheep.

#### Chromosome scale map of sheep *LD* methylome

**Fig 3:**
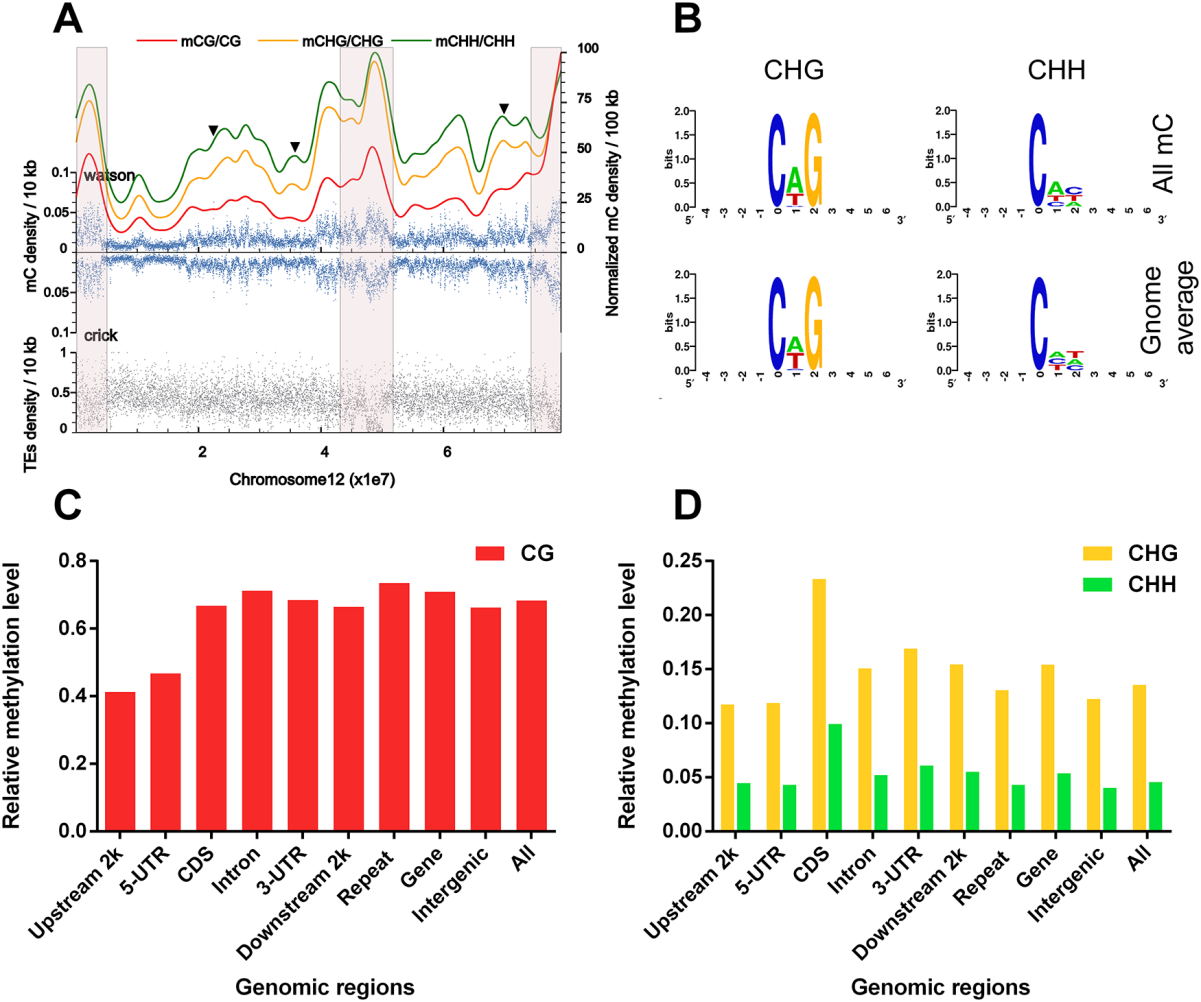
Global DNA methylation trend and distribution. (A) Global DNA methylation trend and repeats density distribution at chromosome scale. X-axis presents length of sheep chromosome 12. Three contexts of mC density at 100 kb windows were profiled in normalized smoothed lines (red line stands for CG context methylation density, yellow line stands for CHG and green line stands for CHH). All mC density at 10 kb windows of both strands was profiled with blue scatter plots and repeats density at 10 kb windows was profiled with gray scatter plots. Black triangles indicate the variant regions that CG methylation shows different with non-CG methylation. Regions highlighted within semitransparent red boxes showed the negative correspond distribution of mC density and repeats density. (B) Logo chart shows the preference of the sequences that proximal to sites with mCHH and mCHG in sheep *LD* muscle methylome. (C) Relative CG methylation level (red bars) of sheep *LD* methylomes in functional genomic regions include upstream 2 kb of gene, 5’UTR, coding sequence (CDS), intron, 3’UTR, downstream 2 kb of gene, repeat sequence, gene body, intergenic region that contains repeats and up & downstream of gene, and all means the whole genome scale. Relative methylation level is calculated as total methylation level divides all considered mC numbers in target region. (D) Relative non-CG methylation levels (green bars for CHG, yellow bars for CHH) of sheep *LD* methylomes in different functional genomic regions.

At the second layer, we profiled chromosome scale sheep *LD* methylation map (Fig 3A and S2 Fig). Both mC density in 10 kb windows (blue plots) from Watson and Crick strands shared the same distribution. Repeats density in 10 kb windows (grey plots) showed a very disordered distribution. However, a handful of regions (especially at edges) of chromosome showed relative high distribution of mC density and opposite peaks for repeats density. This observation is also obtained in genome scale methylome in Fig 2. Additionally, CG methylation (red line, 100 kb window) distribution was different from non-CG methylation at several regions (pointed by black triangles), while CHG (yellow line) and CHH methylation (green line) distributions were highly consistent. Such observations were in accord with that of human methylomes [3].

#### Base resolution scale view of sheep *LD* methylome

Full browsing of sheep *LD* methylome at base resolution could be performed within genome browser tools such like UCSC genome browser or IGV genome browser. Here we exhibit an instance using IGV genome browser [36].

**Fig 4:**
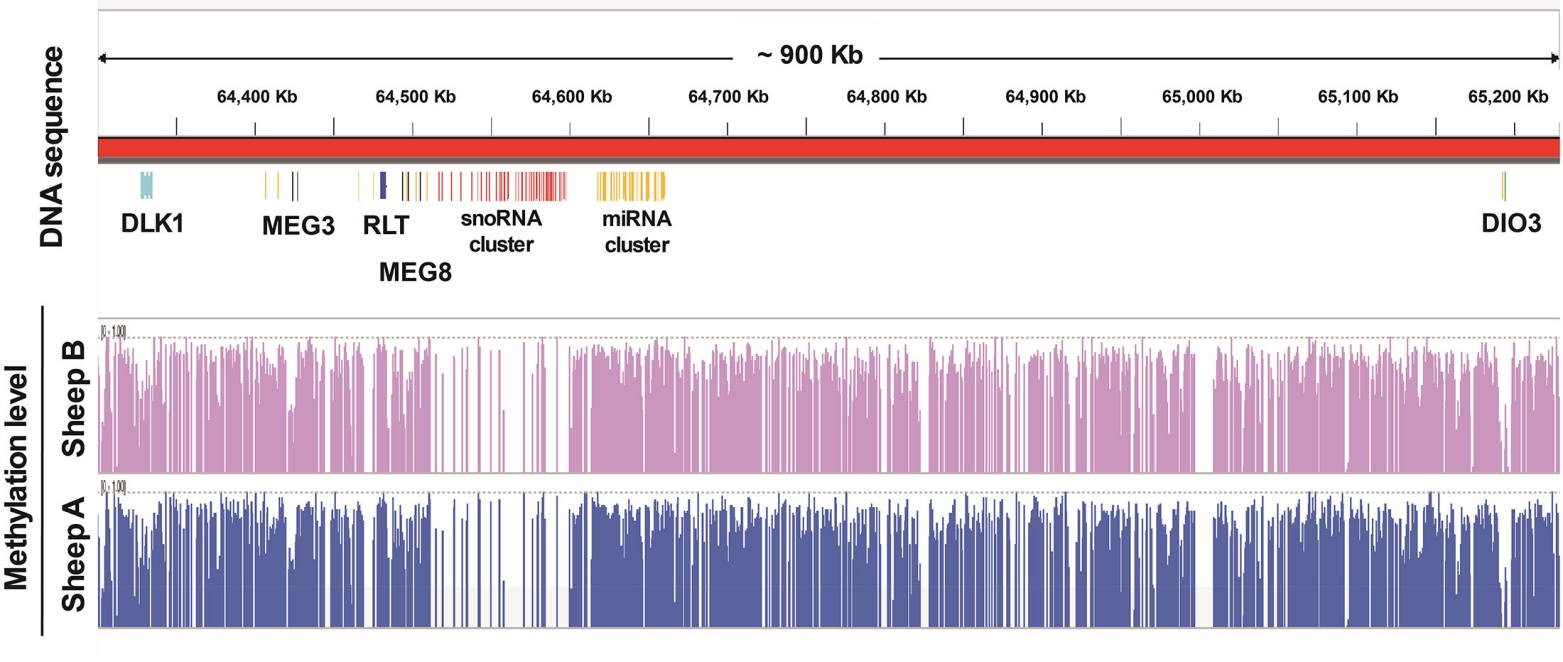
DNA methylation distribution at *Dlk1-Dio3* (Callipyge) region on sheep genome 18 chromosome. Red bar stands for an about 900 kb length genome sequence known as *Dlk1-Dio3* (Callipyge) region. Colorful bands indicate the relative physical location of different genes. Blue bands: *Dlk1*; yellow bands: miRNA; dark gray: *MEG3*; dark blue: *Rlt*; black: *MEG8*; red: snoRNA; green: *Dio3*. At the bottom of figure is CG methylation level histogram chart at base resolution. Blue histogram refers to sheep A, and pink refers to sheep B.

The *Dlk1-Dio3* domain known as representative imprinting control region (ICR) has highly conserved gene order and imprinting status in mammalian [37]. In sheep, a single mutation from A to G somewhere between *Delta-like 1 homologue* (*Dlk1*) and *Maternal Expressed Gene 3* (*MEG3*) gene led to the famous callipyge mutation, which causes a postnatal muscle hypertrophy [38]. Study on such ICR is essential for breeding and helps to accelerate understanding of meat development in sheep. However, base resolution DNA methylation status of *Dlk1-Dio3* in sheep is not studied yet. In this study, we found a similar CG methylation distribution at *Dlk1-Dio3* region of sheep with that reported in human and mouse (Fig 4) [39]. Three protein coding genes *Dlk1*, *Retrotransposon-like 1 (Rtl1)* and *Type 3 Deiodinase (DIO3)*, which are found to be normally paternal expressed, showed CG methylation level notches. Small nucleolar RNA (snoRNA) cluster whose functions remained largely unknown was distinctly low level CG methylated; and the *miRNA-containing gene* (*MIRG*) downstream of snoRNA cluster, showed high CG methylation level as other intergenic regions. In general, our data provides base resolution perspective on particular genomic regions of sheep, which is valuable for geneticist study imprinting cases. This full browsing data set was stored as wiggle (WIG) format, contains all three contexts (CG, CHG and CHH) of DNA methylation status throughout whole sheep genome, and has been uploaded to the *Caprinae* Genome Database (http://caprinae.kiz.ac.cn/index.htm) and formatted as first single base-resolution sheep *LD* methylome database.

Similar to the observations in human and mouse methylomes [19, 40], no nucleotide preference was observed around mCG sites, while A was preferred immediately downstream of mCHG and mCHH sites. Besides, no TA sequence preference was observed at upstream of mCHG and mCHH sites in sheep *LD* methylome (Fig 3B).

### DNA methylation distribution at functional genomic regions

DNA methylation level showed diverse distributions in different functional genomic regions. CG relative methylation level (total methylation level of mC divided by total number of cytosine sites in a region) was overall high, especially in repeat regions, but not in the upstream 2 kb and 5’UTR regions of annotated genes (Fig 3C). CHG and CHH relative methylation levels showed consistent distribution. Different from CG methylation, non-CG methylation showed high levels in coding sequences and relatively low levels at repeat regions (Fig 3D).

**Fig 5:**
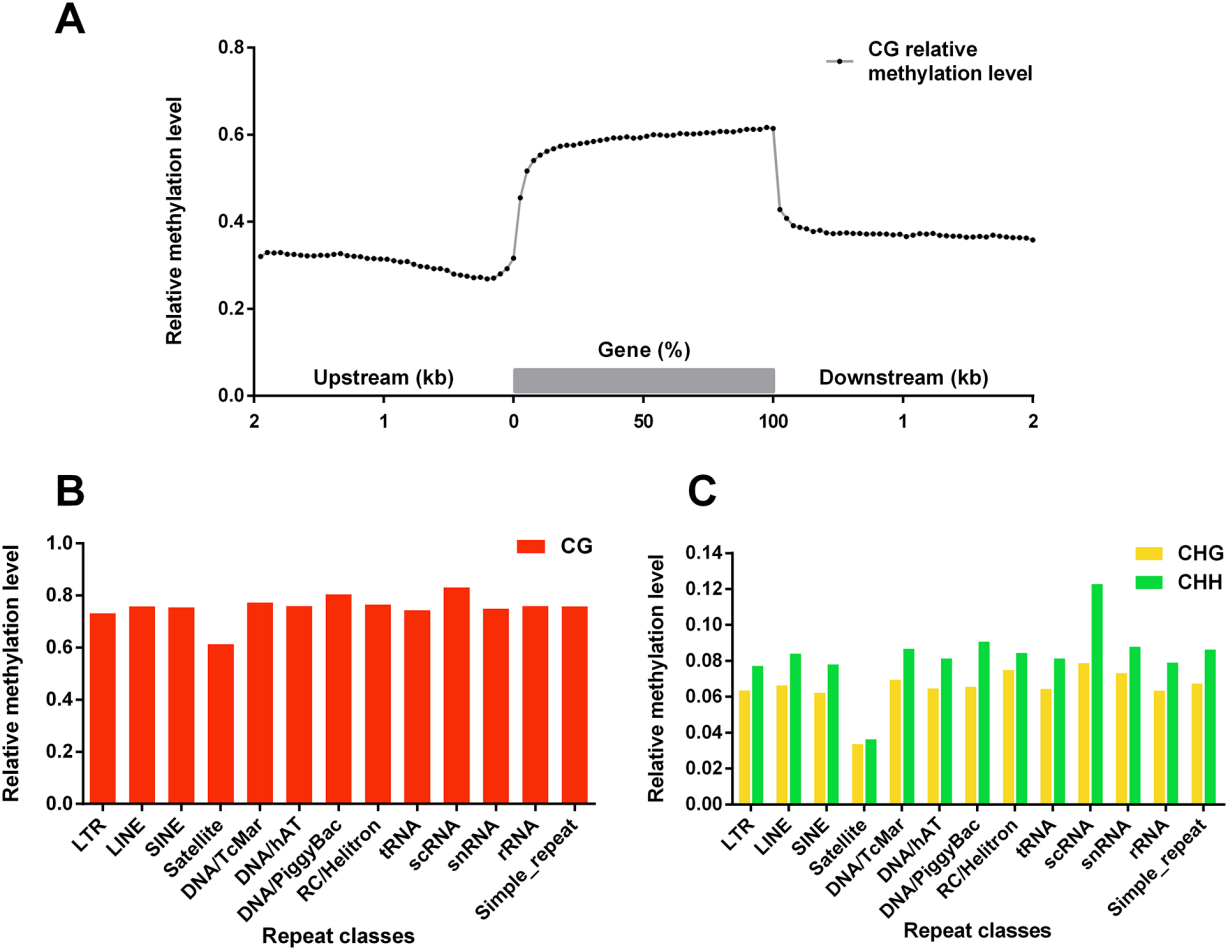
Genes and repeats locus DNA methylation status. (A) Gray line chart indicates relative CG methylation level distribution throughout all annotated genes and their upstream 2 kb and downstream 2 kb regions. A sliding window method was used. 200 bp window size and 100 bp step size were used for flanking DNA sequence. 5% sequence length window size and 2.5% step size were used for gene body regions. (B) Red histogram showed CG methylation status in several categories of repeats. (C) Green and yellow histogram showed CHG and CHH relative methylation level in several categories of repeats respectively.

Comparison of twin sheep methylation patterns revealed high similarity in every functional genomic region. Additionally, CG relative methylation level from Sheep A was a little bit higher than that of sheep B in all categories. Such disparity was not found in non-CG methylation. Since CG methylation was thought to be involved in expression process, a sliding window approach was carried out to profile variations of CG relative methylation level around gene locus (Fig 5A). In gene body, CG relative methylation level is obviously higher than flanking regions. 5’ upstream regions showed lower relative methylation level than 3’downstream region. All of these distributions were observed similar with recent studies in horse and silkworm methylomes [41, 42]. Besides, relative methylation levels in several categories of repeats were also described (Fig 5B and 5C). CG relative methylation level showed high level in almost all repeats categories, implying the important role (of what) in repressing transposable elements and maintaining genome structure stability [43]. Interestingly, the highest methylation level was observed in small cytoplasmic RNA (scRNA), which is the component of signal recognition particle, in targeting presecretory proteins to the endoplasmic reticulum membrane [44]. The low relative methylation level in satellite DNA could be partially due to the special base composition of satellite DNA, since satellite DNA is known as having extreme preference of bases. The relationship between DNA methylation and all kinds of repeats sequence in mammalian needs more studies.

### DMR identification between two sheep *LD* methylomes

DNA methylation contributes to various phenotypes via regulating gene expression [45]. Since CG methylation was broadly considered to be involved in some gene regulation processes in mammals, we focused on those CG context DMRs that locate in, or besides the gene locus. All annotated genes from sheep reference genome were involved in study and were parted into 6 categories include gene upstream 2 kb, 5’-UTR, intron, coding exon, 3’-UTR, gene downstream 2 kb. DMRs locate in such regions were supposed to impact gene expression. Software swDMR was used to identify DMRs throughout the whole sheep genome. Strict parameters were set to run swDMR in a conservative environment. 948 DMRs belonging to 248 genes were identified and listed in S1 Table.

### Analyses of DMR-containing genes

For indicating potential roles of these DMR-containing genes, InterProScan 5 software was used to perform Gene ontology annotation. 14250 of all 22881 sheep annotated genes have at least one GO annotation. A gene over representative tool called Ontologizer was used to perform statistical enrichment analysis of gene set [28]. A total of 129 GO terms were significantly enriched (p < 0.05), and listed in S2 Table. In addition, KEGG annotation and enrichment analysis of such DMR-containing genes were also profiled using KOBAS 2.0 [46]. KEGG annotation analysis showed significantly (P< 0.05) enriched DMR-containing genes in four pathways (Table 3). Interestingly, six DMR-containing genes in the progesterone-mediated oocyte maturation and estrogen signaling pathways were enriched in KEGG analysis, suggesting a link to the time of sexual maturity. Considering sexual maturity time of female Sunit sheep is at the age of 6 to 7 months and is deeply impacted by growth environments. We speculated that 6 DMR-containing genes clustered to such pathways might be because one of twin sheep had earlier sexual maturity than the other, due to different habitats. Four DMR-containing genes were KEGG enriched in term “transcriptional misregulation in cancer”. Besides, RAS signaling pathway and “transcriptional misregulation in cancer” term were also observed to enrich 10 DMR-containing genes, while the consensus finding is that 2 DMR-containing genes in “protein-arginine deiminase activity” were GO enriched (very significantly enriched with P < 0.0015). Since protein-arginine deiminase is known as the tumor repression production, such observations suggested a tumor-related difference within *LD* muscles between twin individuals. Checking GO enrichment results, we found that DMR-containing genes related to lipid and fatty acid oxidation were significantly GO enriched (P < 0.047). Considering DNA methylation has a role in gene expression regulation, these observations could be molecular clues or witnesses of phenotypic variant that twin sheep had big different in weight.

**Table 3:** KEGG annotation and significantly enrichment terms of DMR-containing genes.

Terms	DMR-containing genes number	Background genes number	P value	Gene name
**cAMP signaling pathway**	5	216	0.029	CCG022955.0, CCG003638.0, CCG013269.0, CCG000672.0, CCG002906.0
**Transcriptional misregulation in cancer**	4	168	0.045	CCG017984.0, CCG014717.0, CCG014844.0, CCG001521.0
**Progesterone-mediated oocyte maturation**	3	93	0.039	CCG003834.0, CCG004983.0, CCG003638.0
**Estrogen signaling pathway**	3	102	0.048	CCG014871.0, CCG004983.0, CCG003638.0

## Discussion

In this study, we presented the sheep *LD* methylome at single base resolution with high coverage from the muscle tissues of dizygotic twin Sunit sheep, which helps elaborate DNA methylation pattern of major economic mammals. We observed DNA methylation predominantly occurred at CG dinucleotide sites. However, we also found non-CG methylation in sheep *LD* methylome occupied more than half of methylated cytosines. Non-CG methylation level was very low, which might result from the diverse cell types from sheep muscles. Importantly, whole genome distribution of non-CG methylation density was observed to be consistent with CG methylation. In addition, sequence preference around mCHH or mCHG also showed similar result with hESC methylome and mouse frontal cortex methylome [3, 23]. These observations well support the existence of non-CG methylation in sheep *LD* methylome.

We described both CG and non-CG methylation status in functional genomic regions like annotated genes or repeats sequences and compared these results with that observed in different mammalian cell types and tissues [3, 40, 41, 47]. Along with those early studied mammalian methylomes, our data provided potential opportunity to deeply reveal function of DNA methylation in variety species.

The methylomes of the twin sheep were very much similar, suggesting high stability of DNA methylation during the 6 months. Additional analyses on specific functional genomic regions showed supporting evidence.

948 DMRs within 248 genes was identified between two sheep samples, which constituted only 1% of whole sheep annotated gene set. We observed from GO and KEGG enrichment analyses that DMR-containing genes were significantly enriched in the lipid metabolism pathway. This finding corresponds to the weight and stature of the twin Sunit sheep. Besides, several genes were enriched in the reproduction related pathways. Time of sexual maturity is important for animal breeding, which can also be impacted by the environment of growth. However, it is not clear whether DMRs are a cause or effect of specific gene transcription regulation. Regardless of the potential influence of methylome inheritance from parents, we conclude that DNA methylation is relatively stable between twin individuals, and variations may be derived from different environmental factors.

## Supporting Information

S1 Fig Three contexts of mC density from twin sheep maps at genome scale. (A) Line chart of repeats density in 100 kb windows. (B) Normalized mCG density (Broken lines indicate normalized methylated cytosine density in CG context in 100 kb windows. Red line refers to sheep A while blue line refers to sheep B). (C) Normalized mCHG density. (D) Normalized mCHH density. (E) Oar v3.1 chromosome bars. Highlighted gray bands refer to normal annotated genes while red bands refer to genes that contain DMRs.
(PDF)

S2 Fig The density of mC and the density of repeats maps profiled throughout whole sheep genome. X-axis presents length of sheep chromosomes. Three contexts of mC density at 100 kb windows were profiled in normalized smoothed lines (red line stands for CG context methylation density, yellow line stands for CHG and green line stands for CHH). All mC density at 10 kb windows of both strands was profiled with blue scatter plots and repeats density at 10 kb windows was profiled with gray scatter plots.
(PDF)

S1 Table DMR-containing genes list. (XLS)

S2 Table Significantly enriched GO terms. (XLS)
