# Supplementary figures and images for: Single Base-Resolution Methylome of the Dizygotic Sheep
Source: PLoS One. 2015 Nov 4;10(11):e0142034. doi: 10.1371/journal.pone.0142034 (PMC4633158; doi:10.1371/journal.pone.0142034)

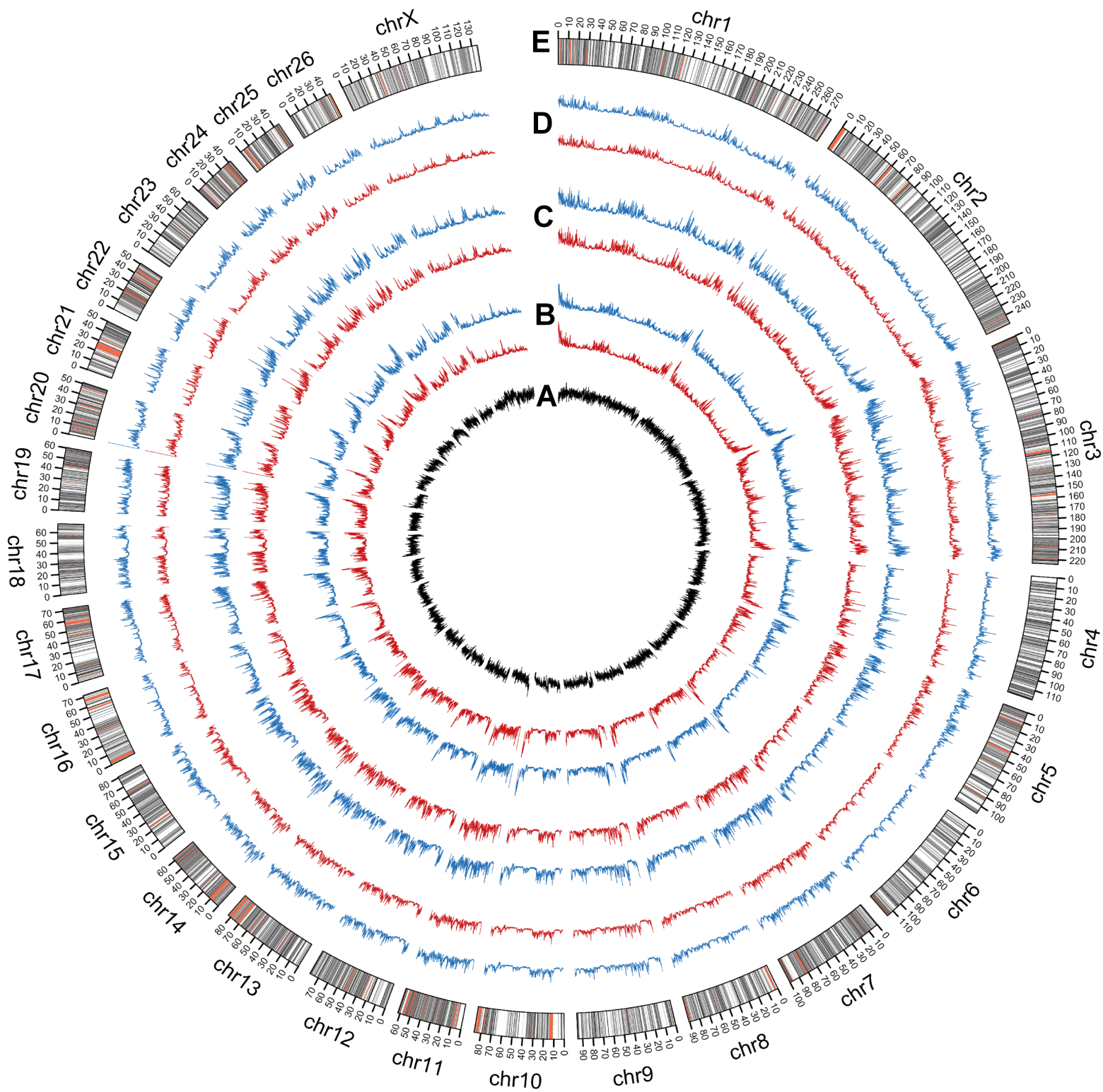

Supplement: S1 Fig — (A) Line chart of repeats density in 100 kb windows. (B) Normalized mCG density (Broken lines indicate normalized methylated cytosine density in CG context in 100 kb windows. Red line refers to sheep A while blue line refers to sheep B). (C) Normalized mCHG density. (D) Normalized mCHH density. (E) Oar v3.1 chromosome bars. Highlighted gray bands refer to normal annotated genes while red bands refer to genes that contain DMRs. (PDF) [file pone.0142034.s001.pdf]

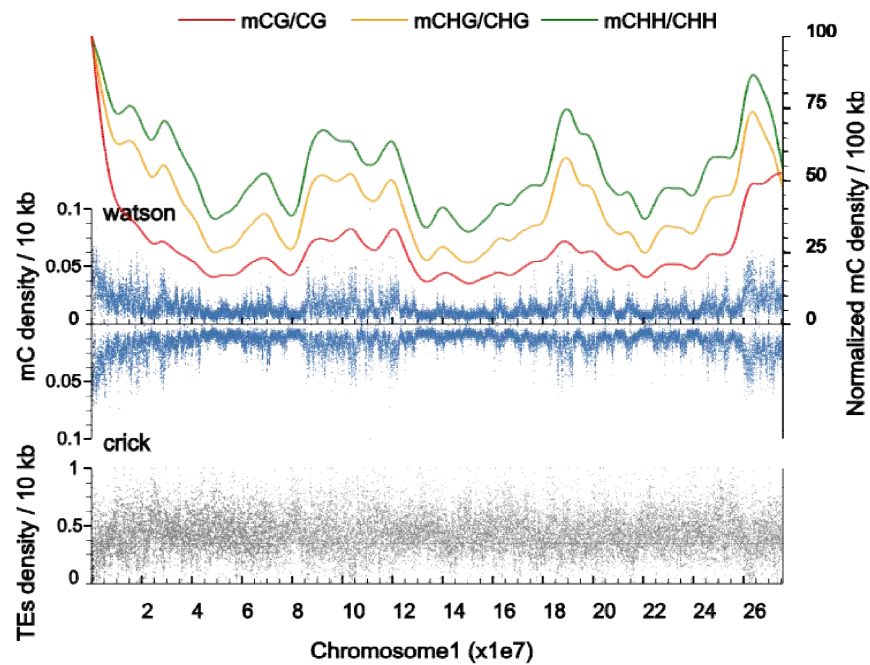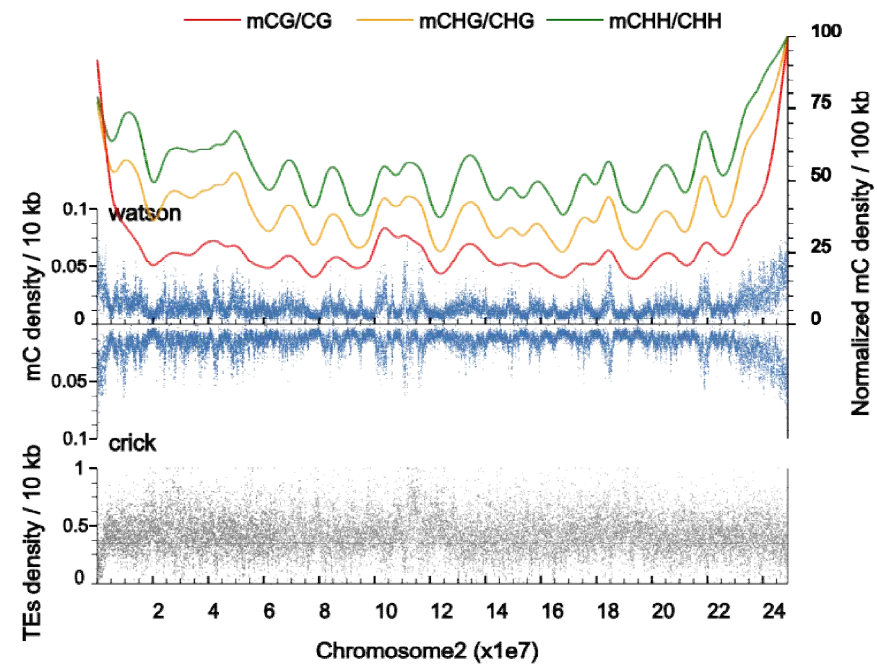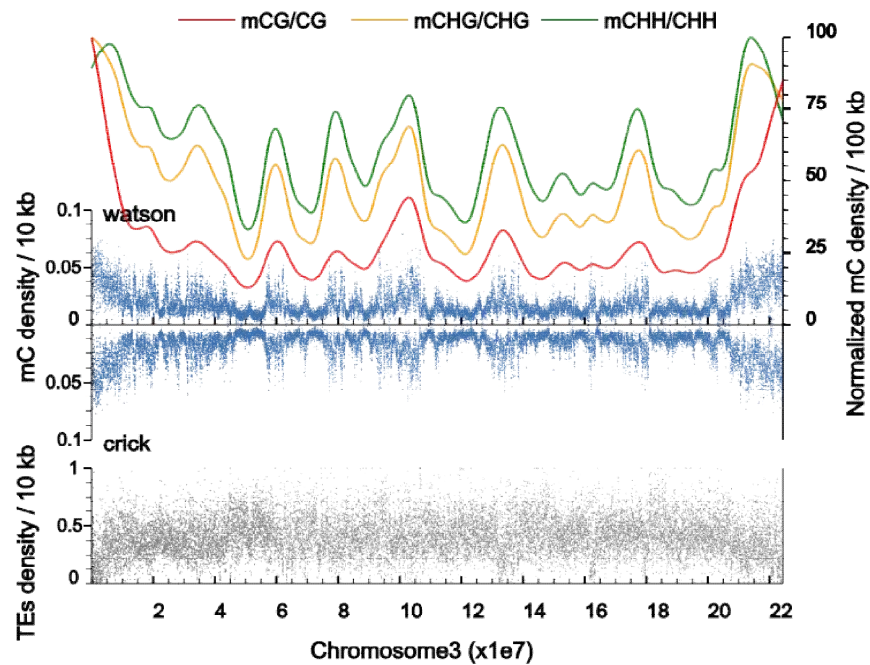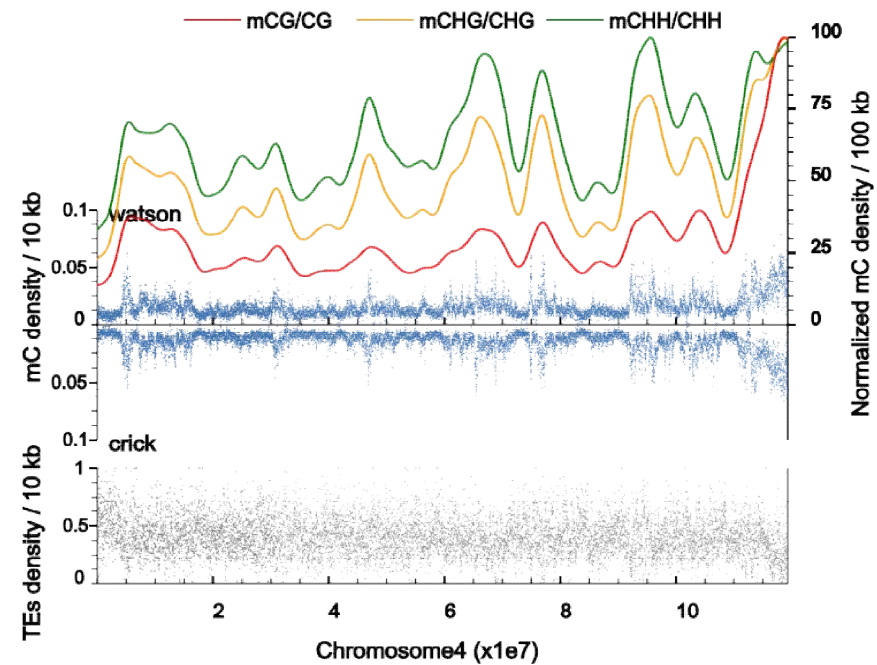

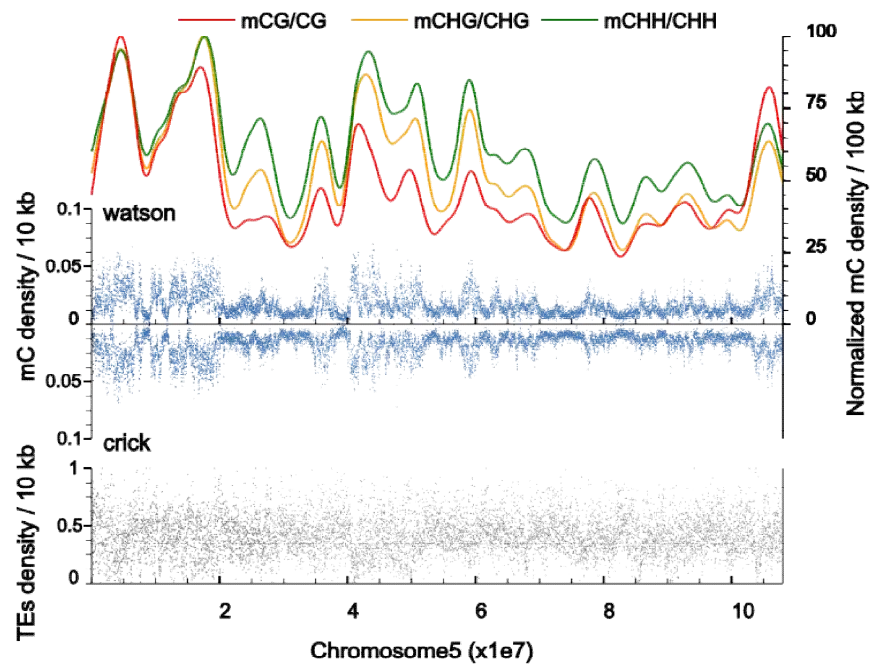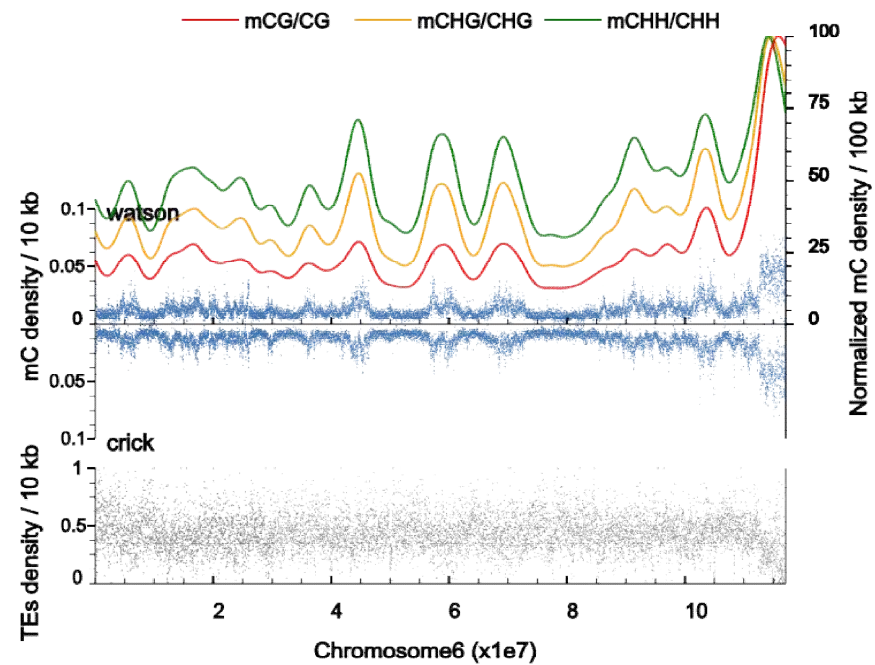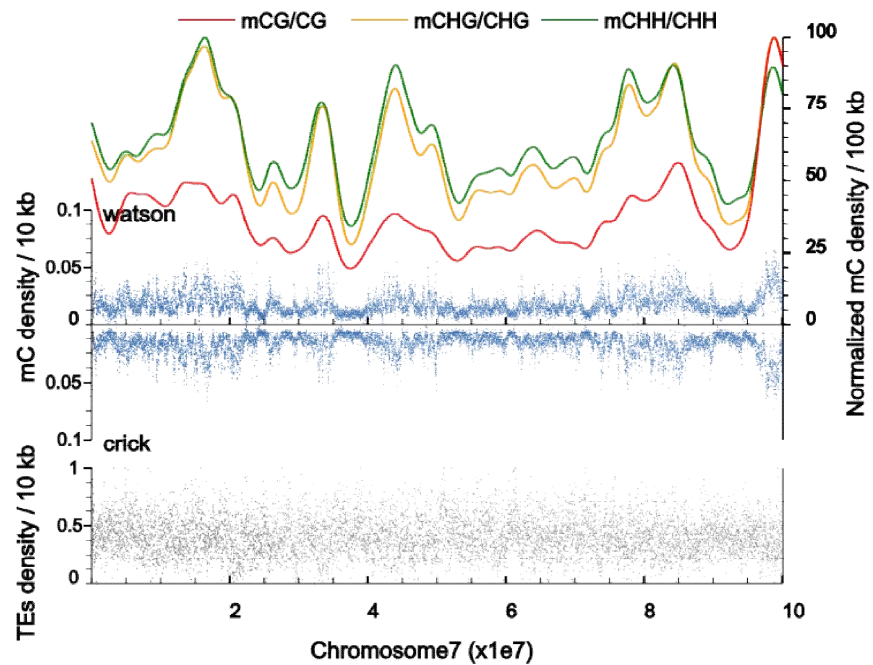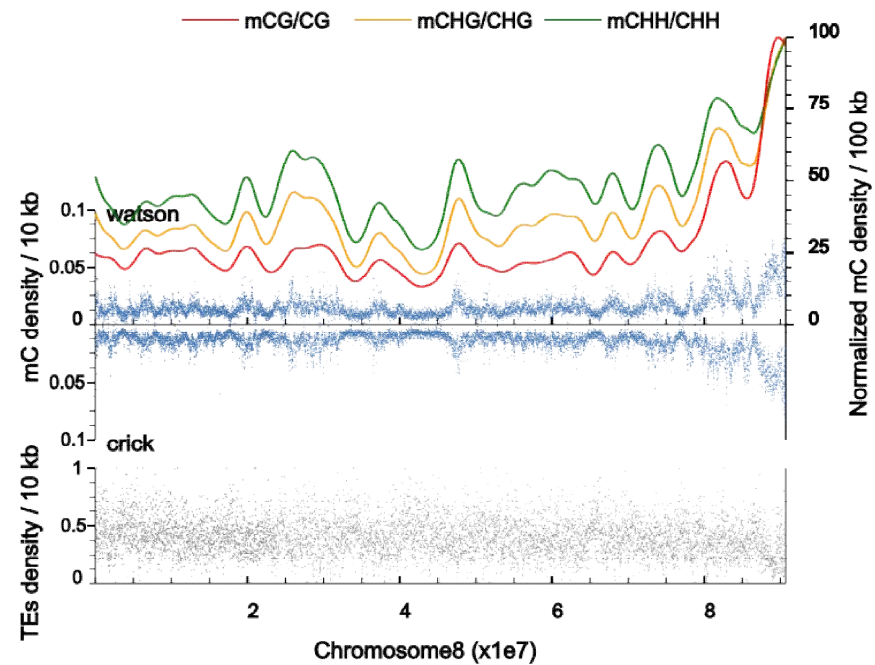

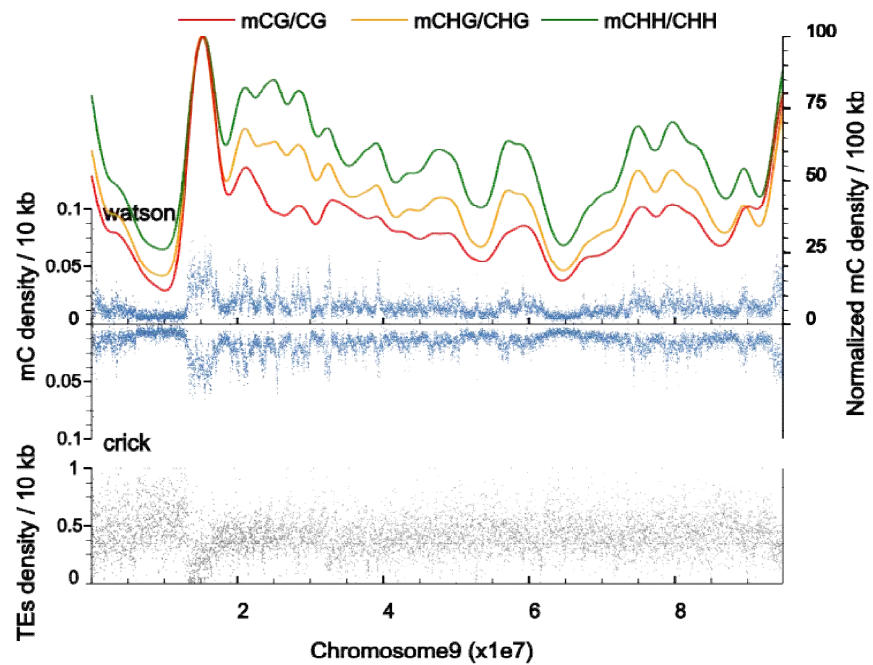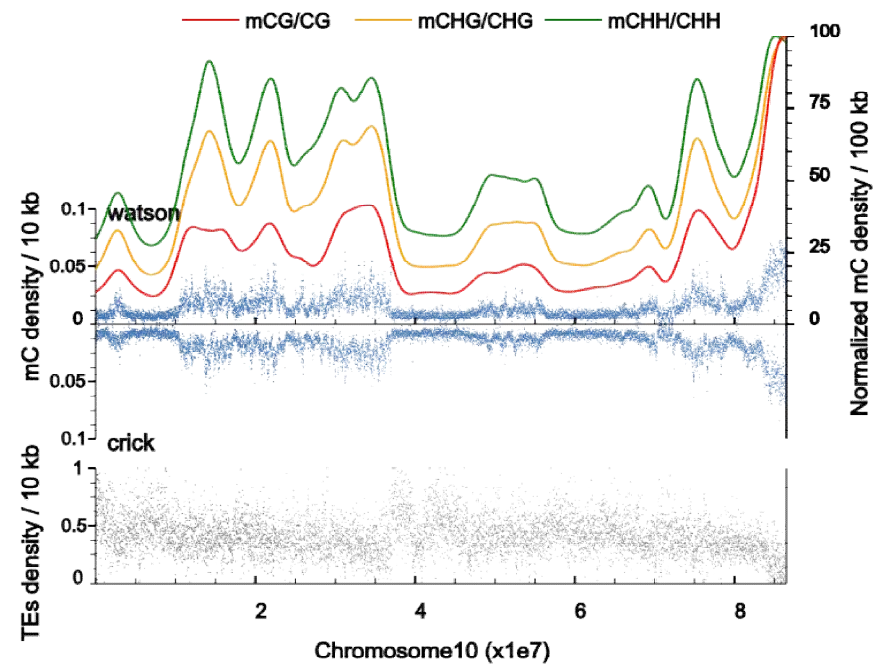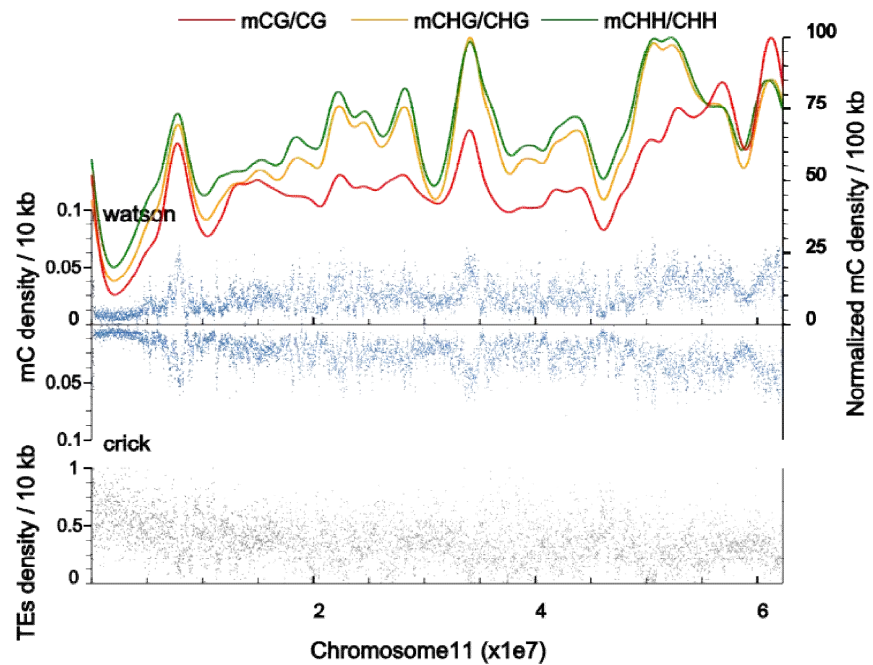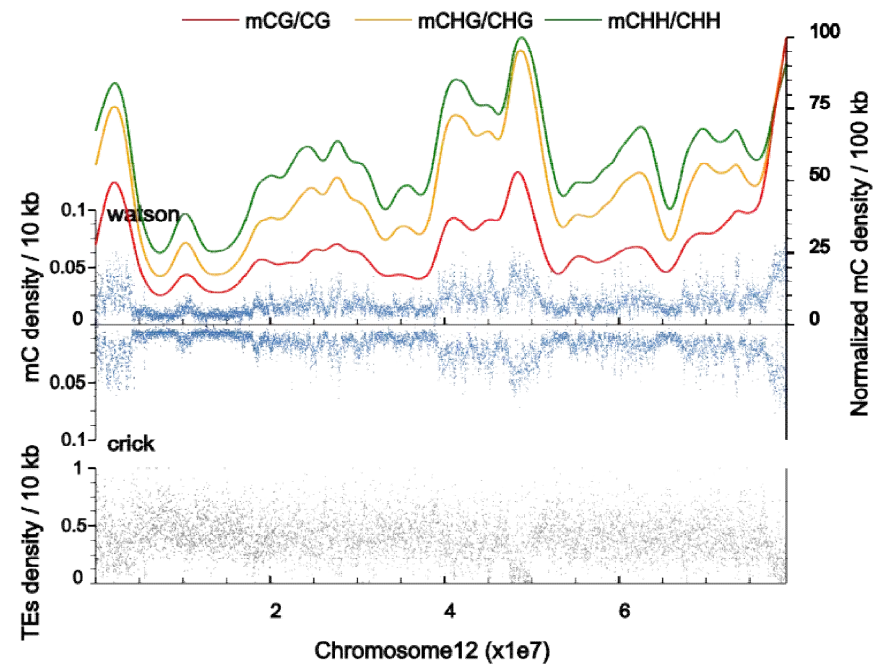

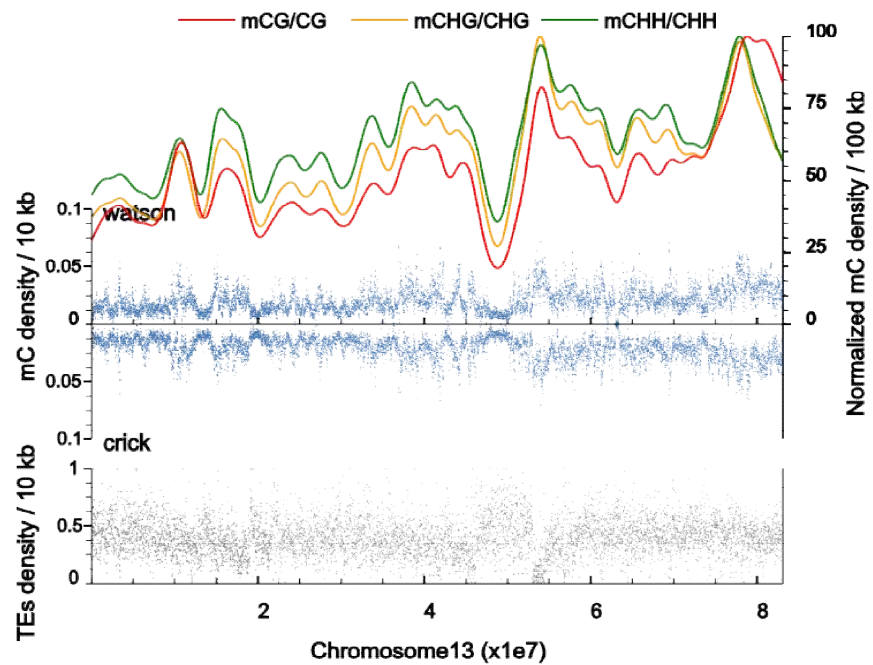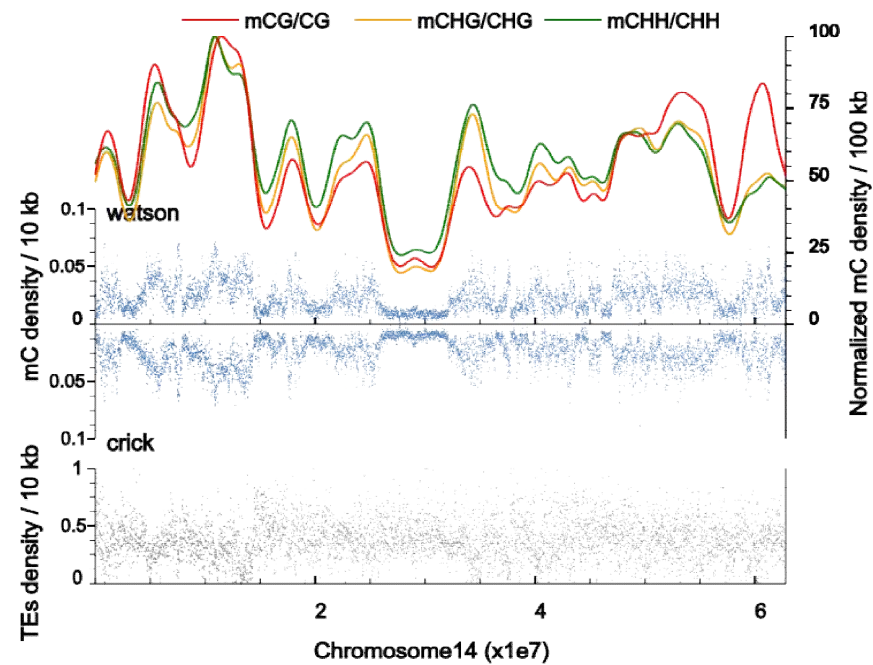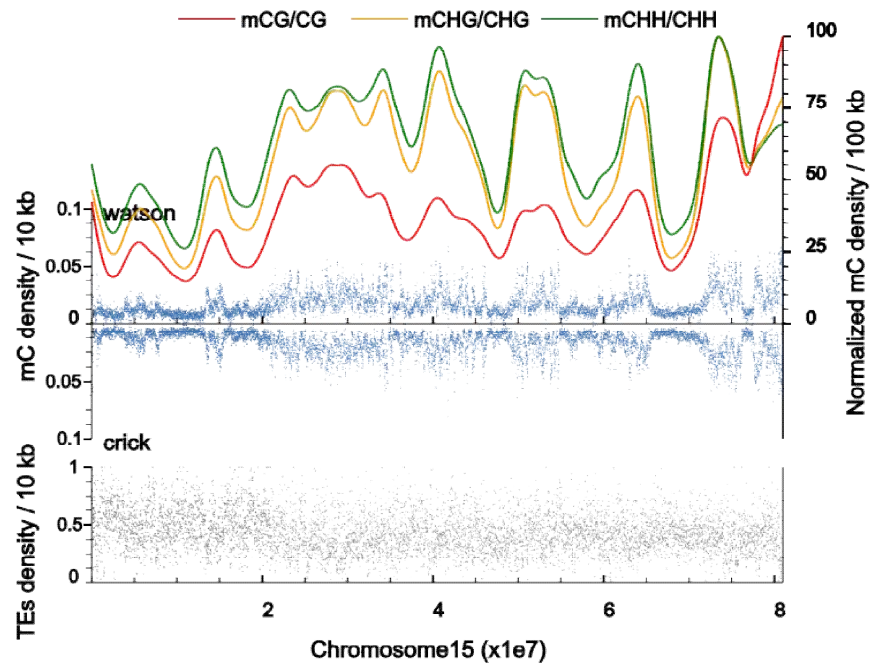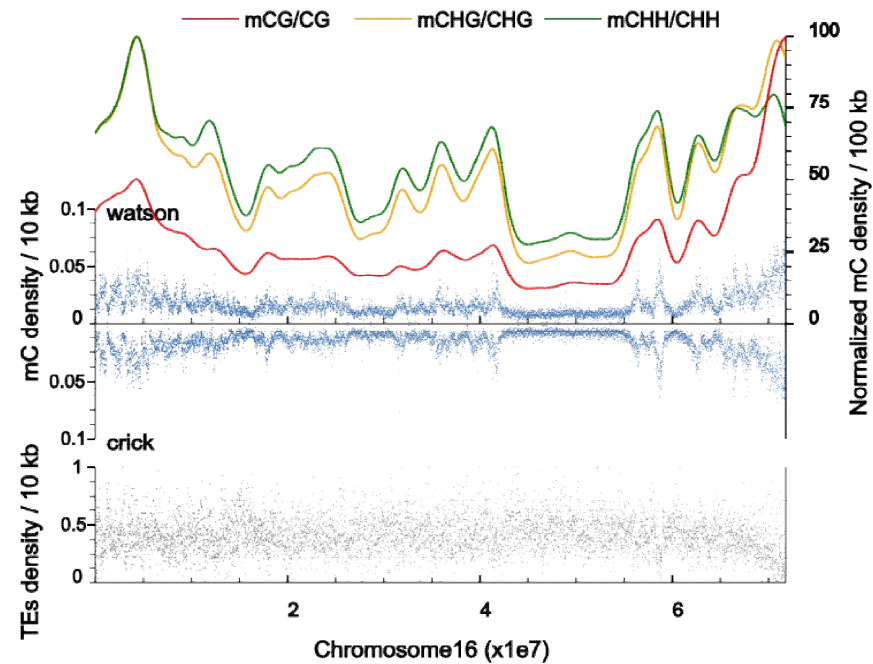

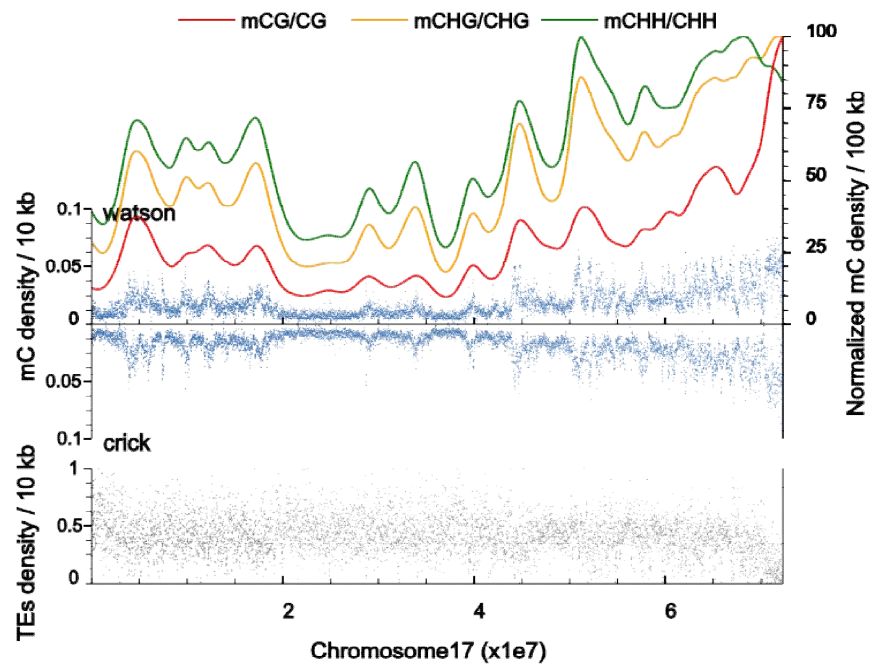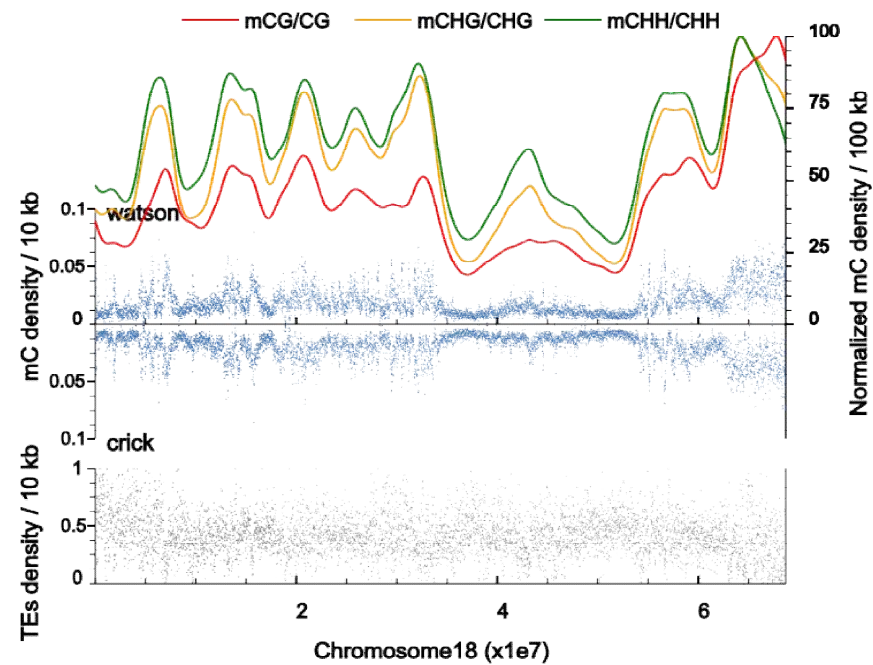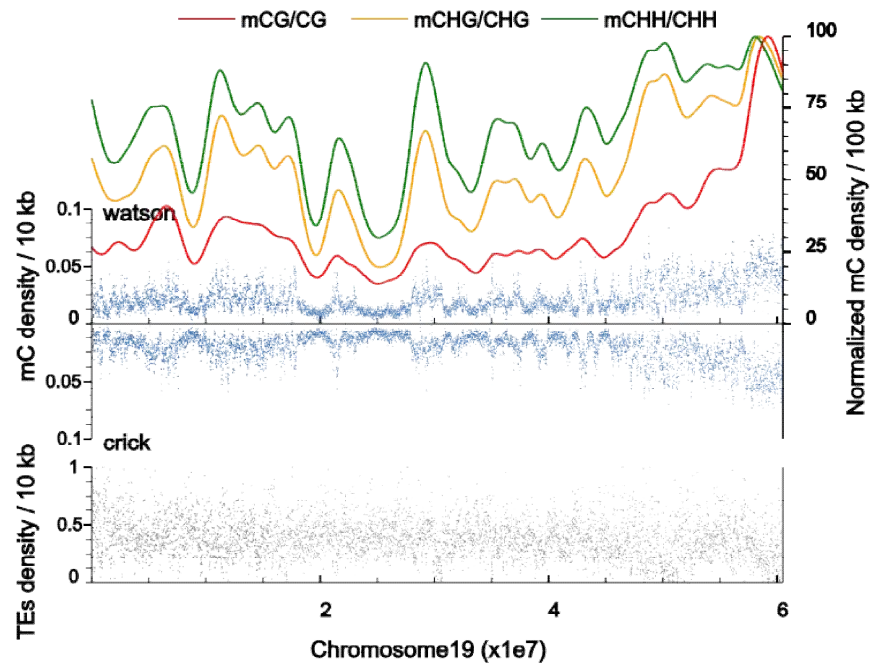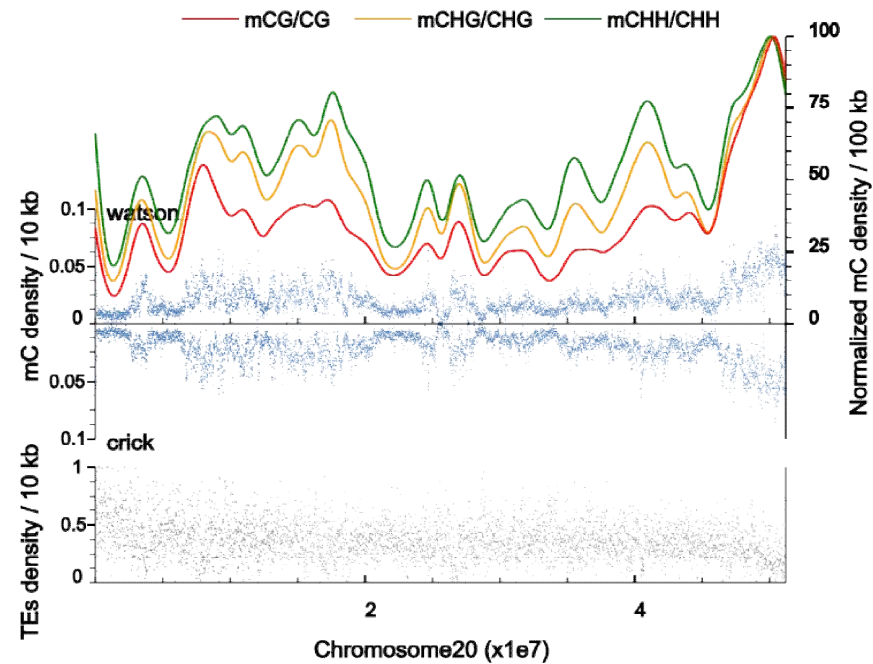

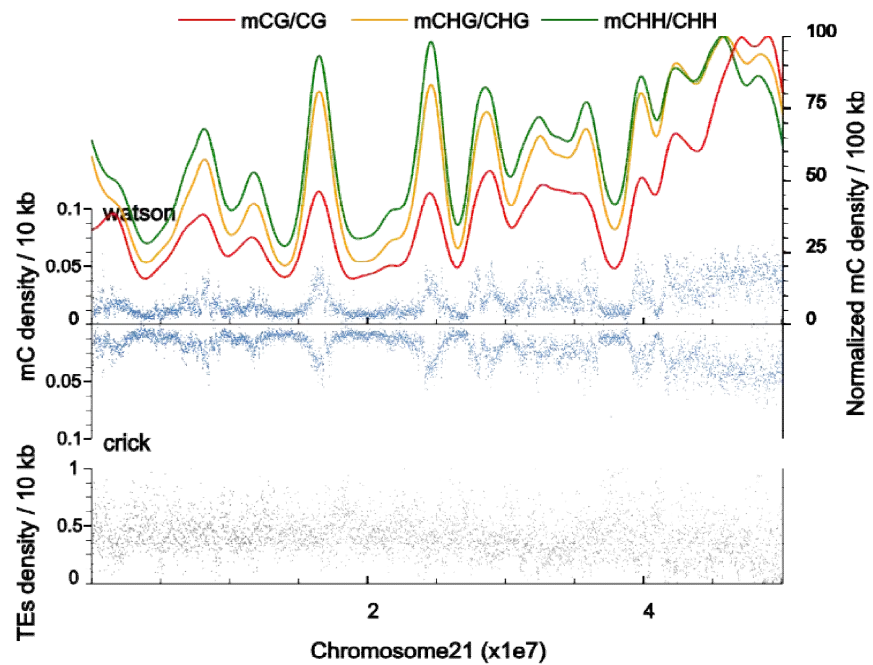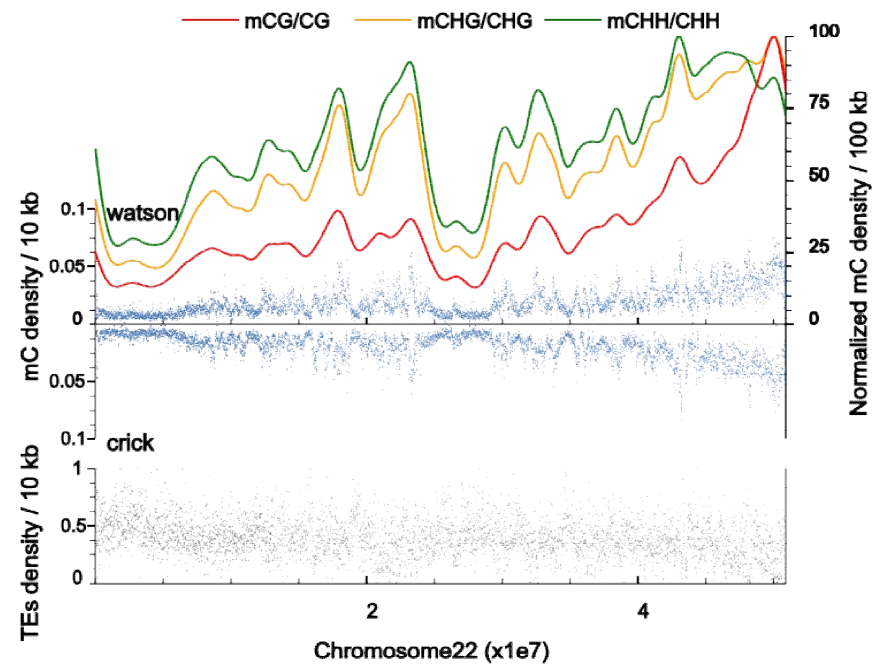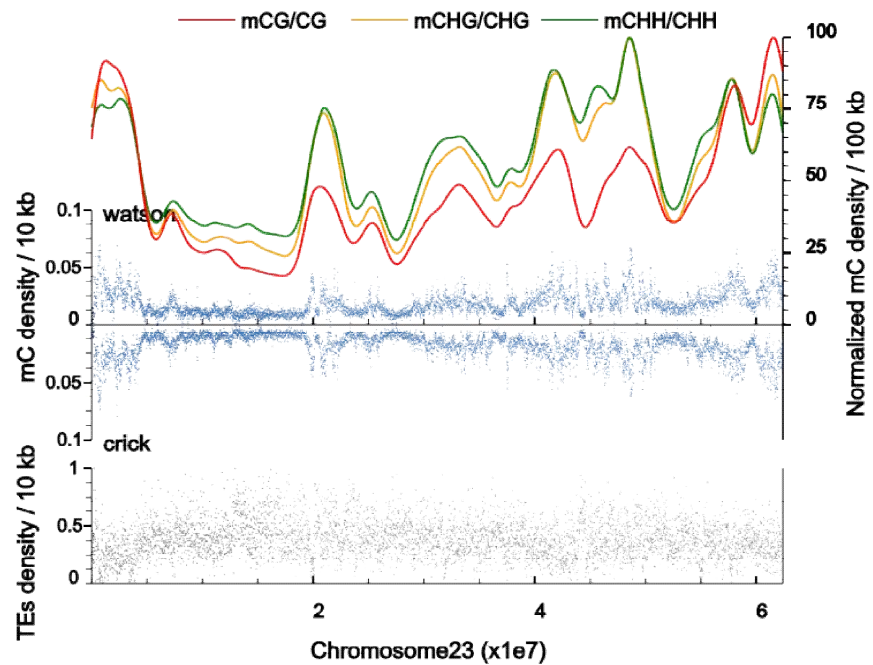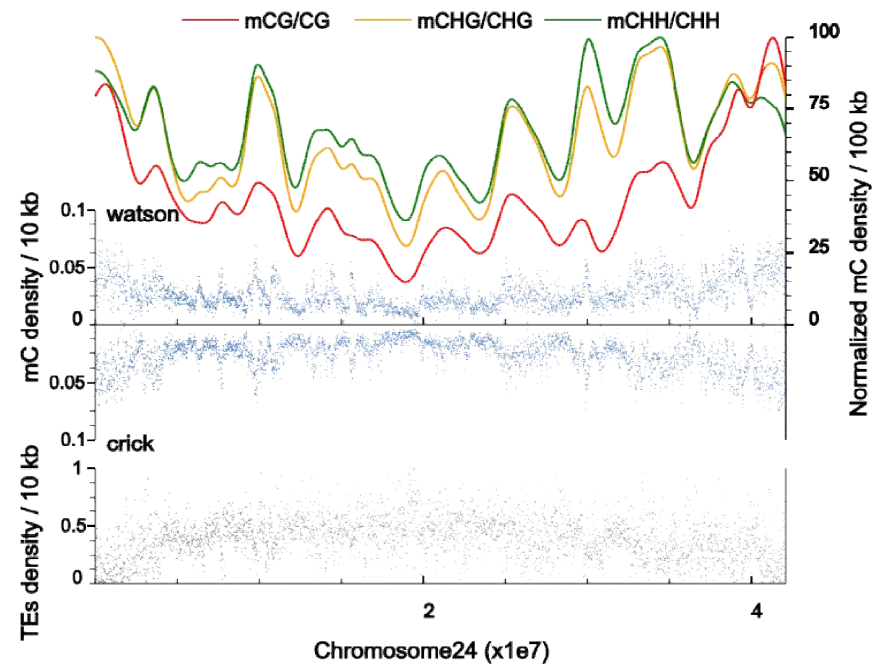

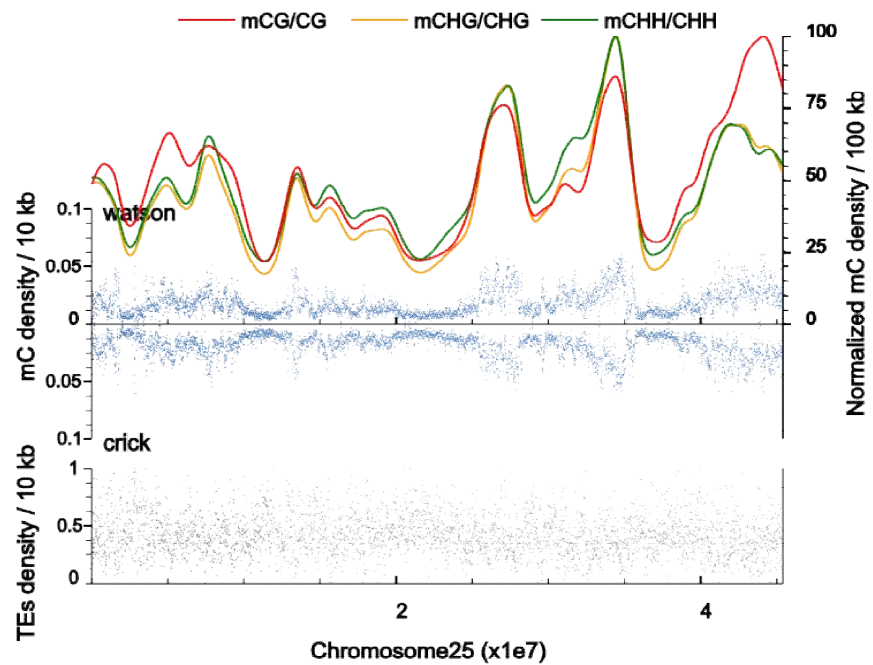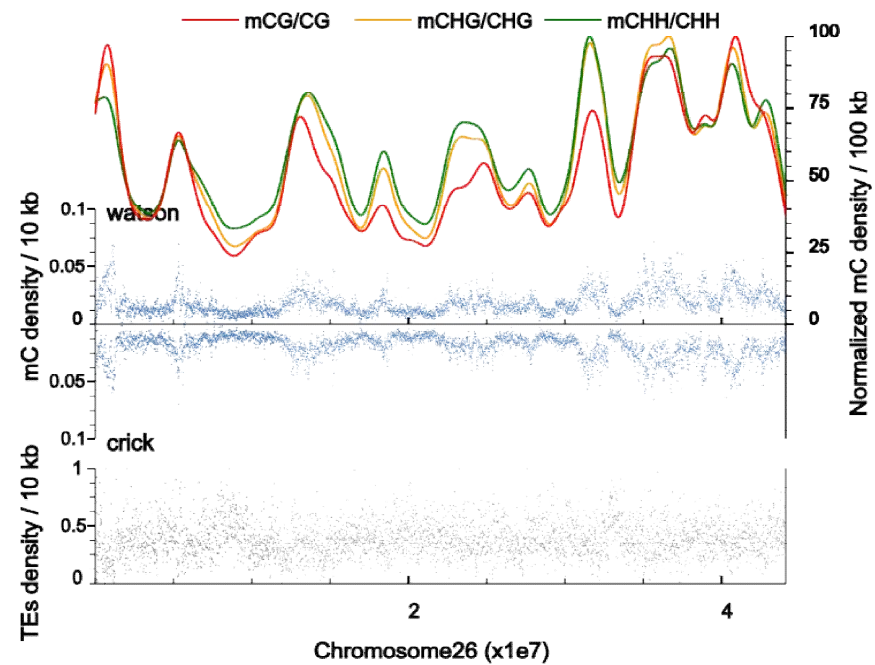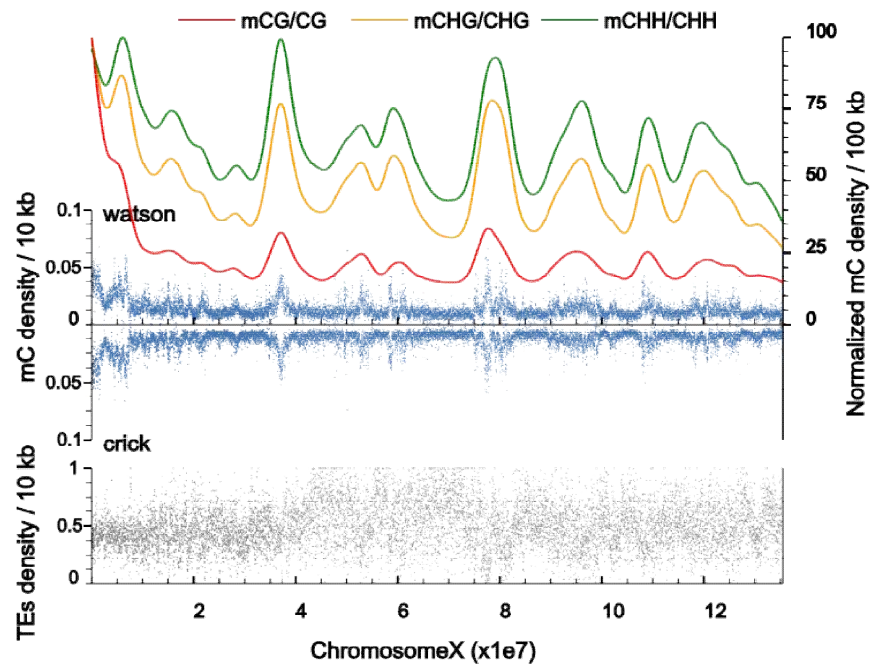

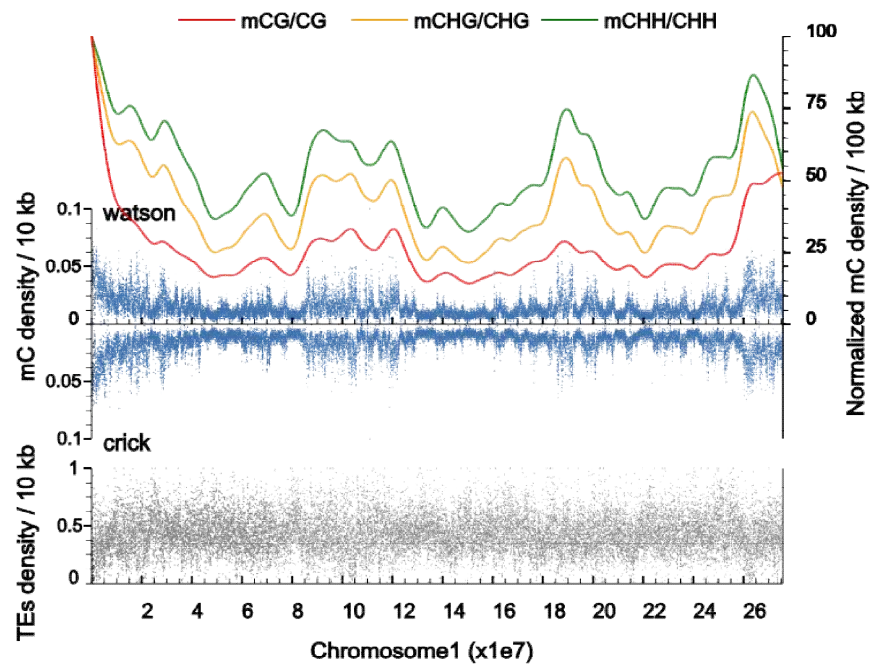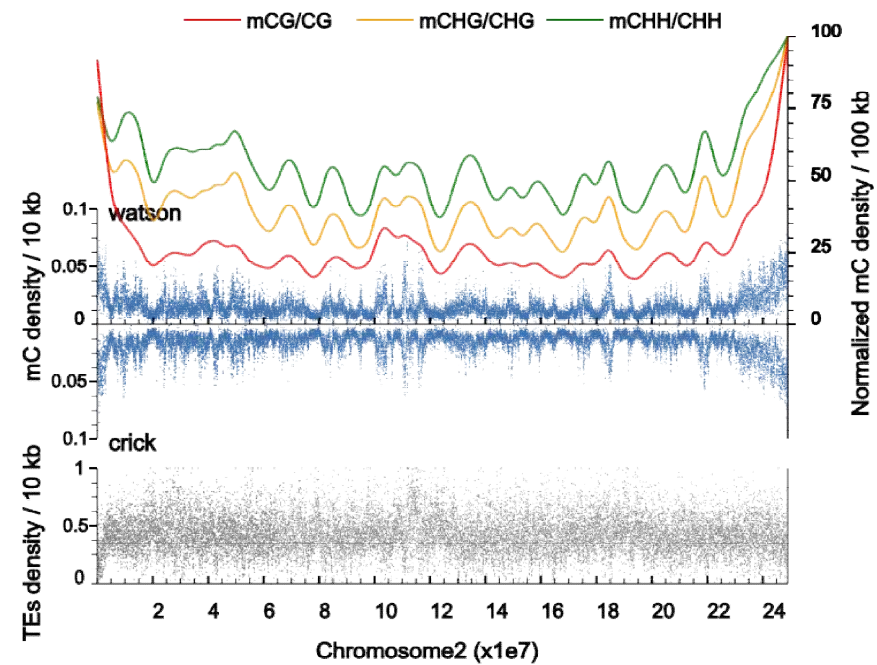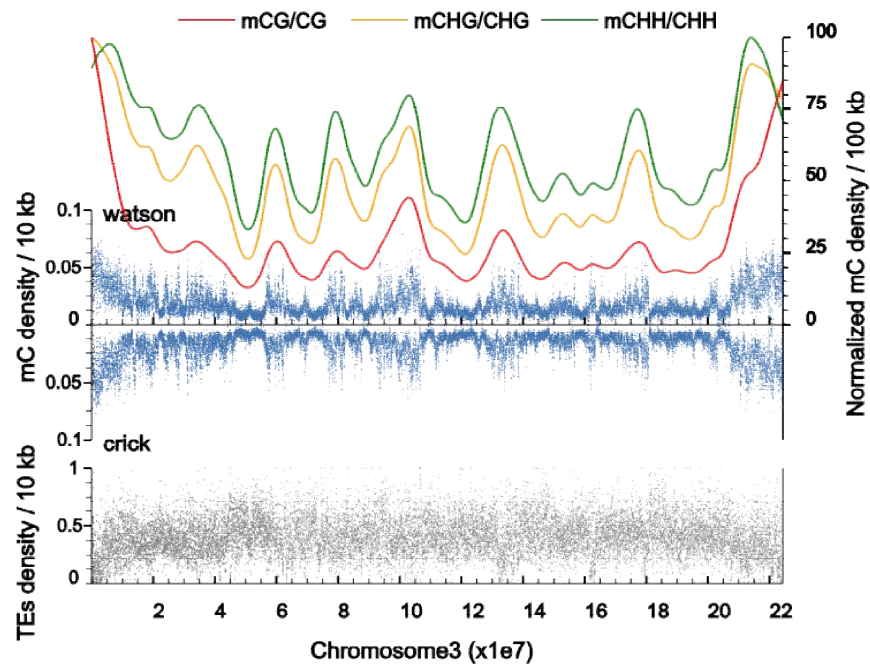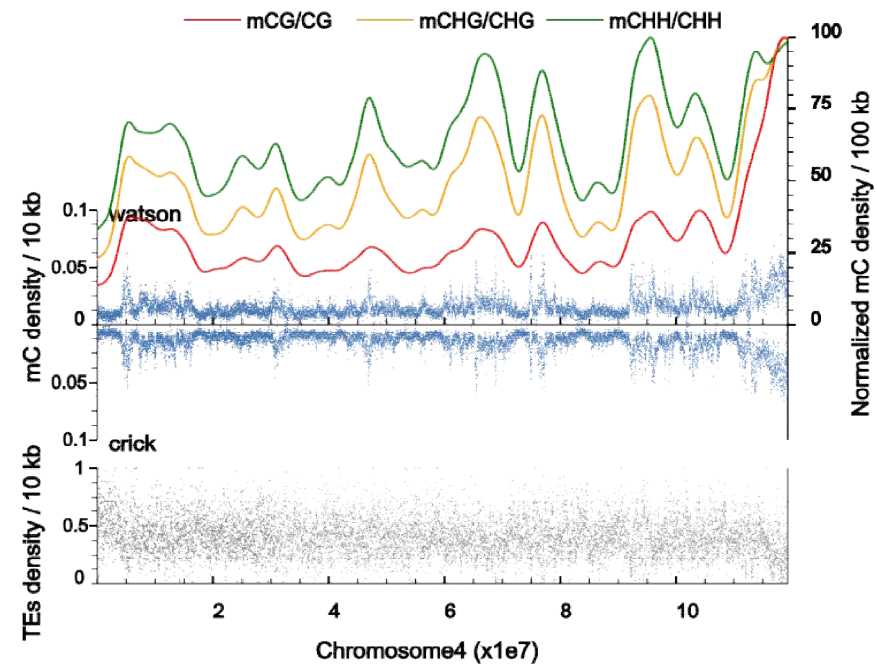

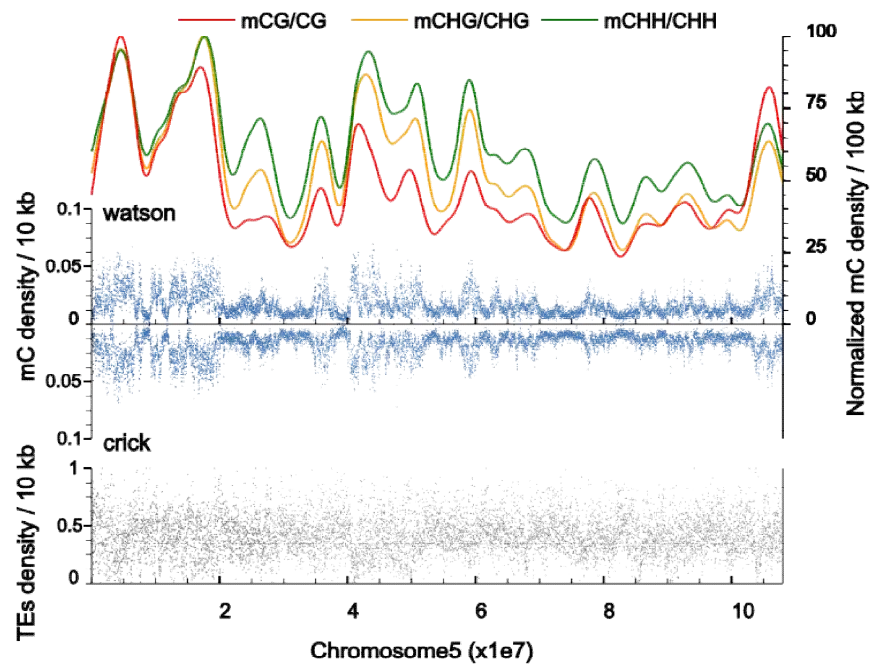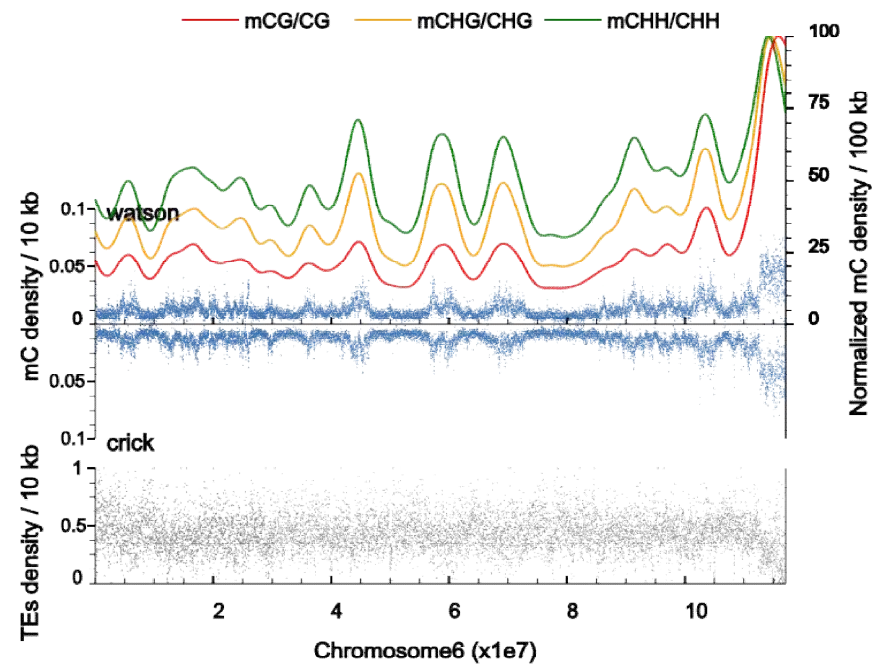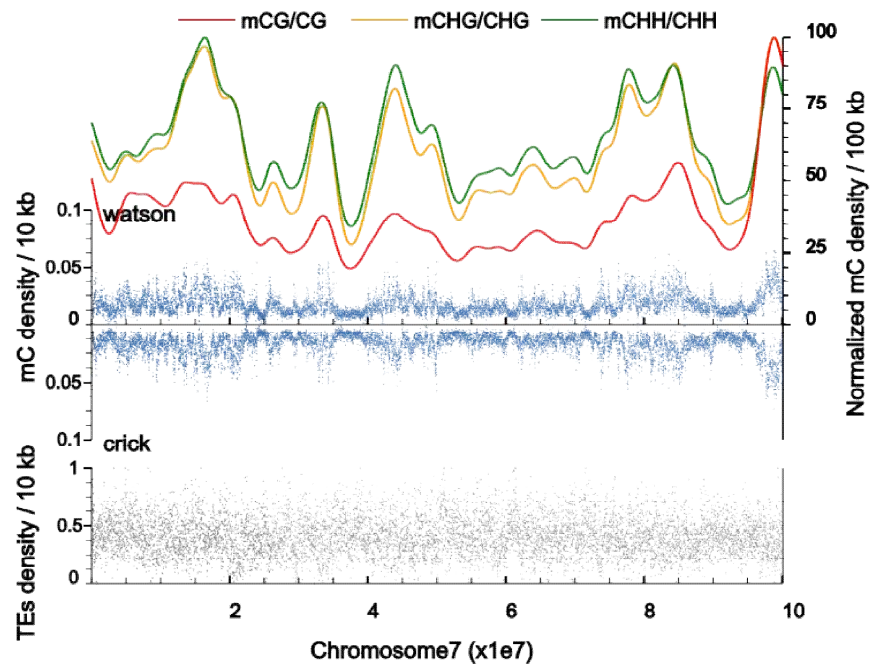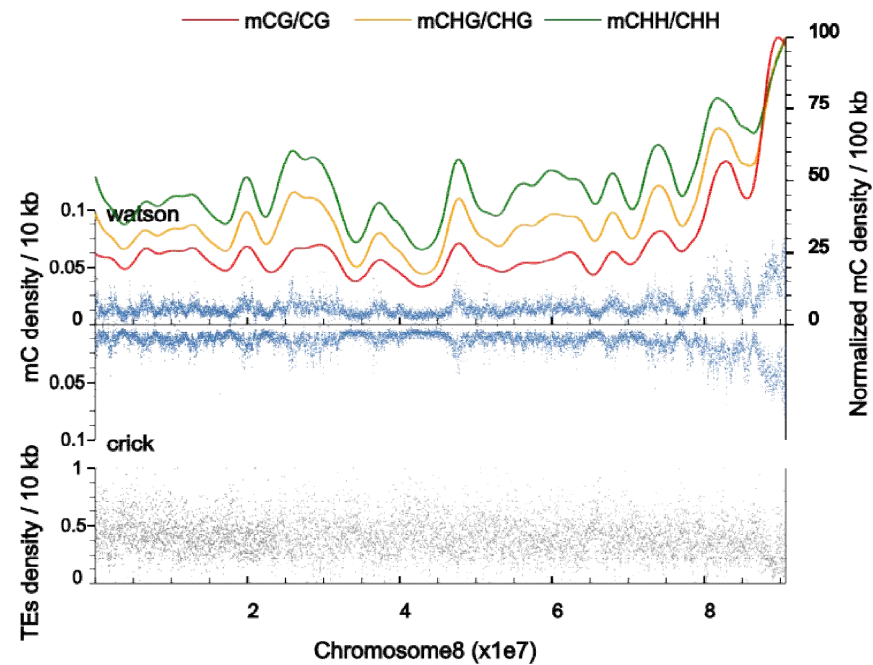

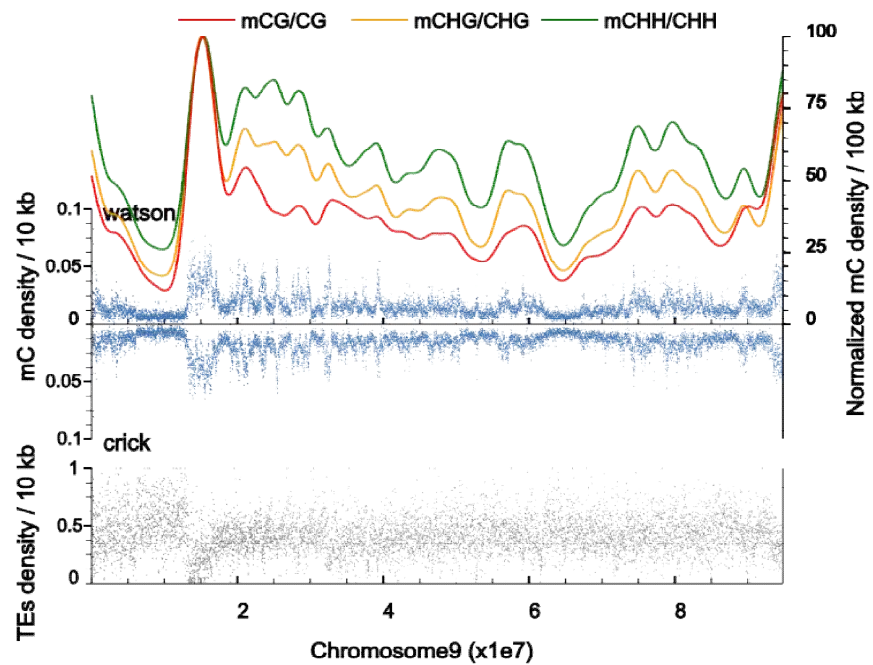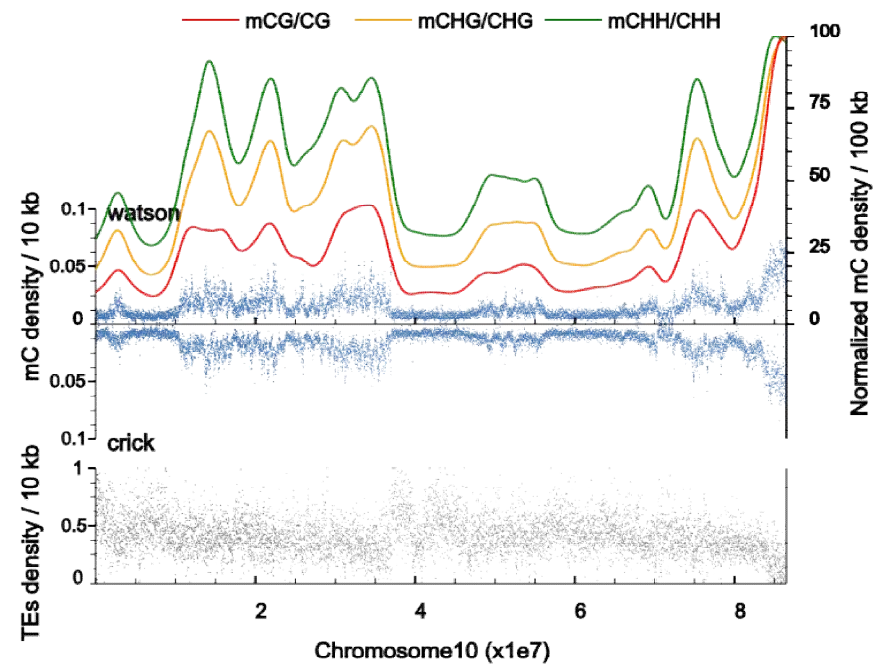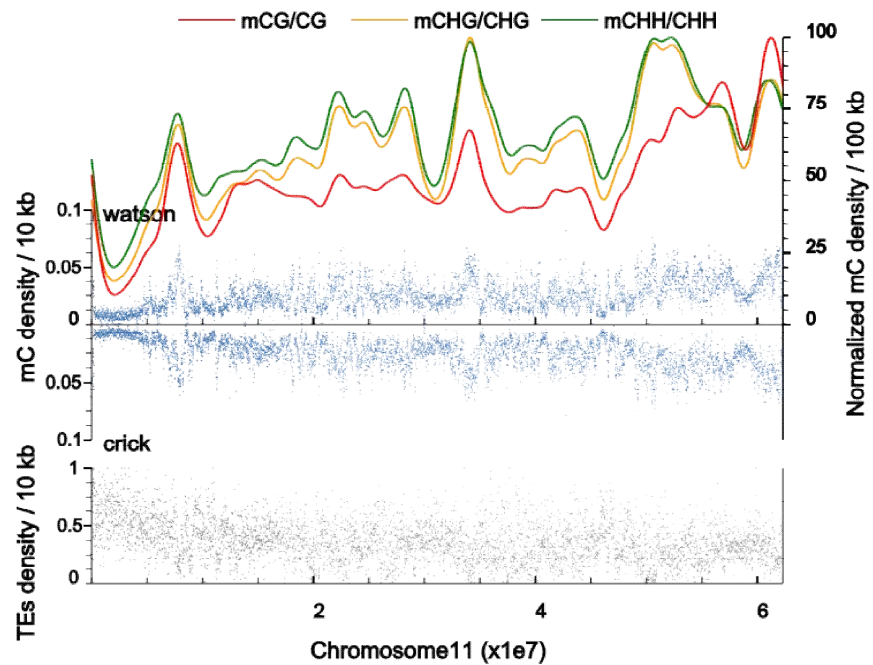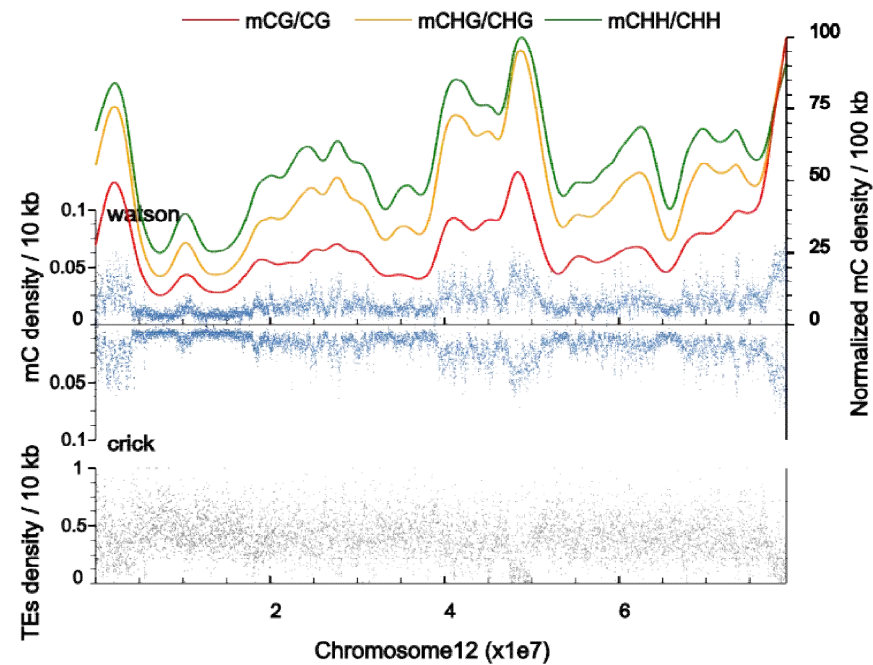

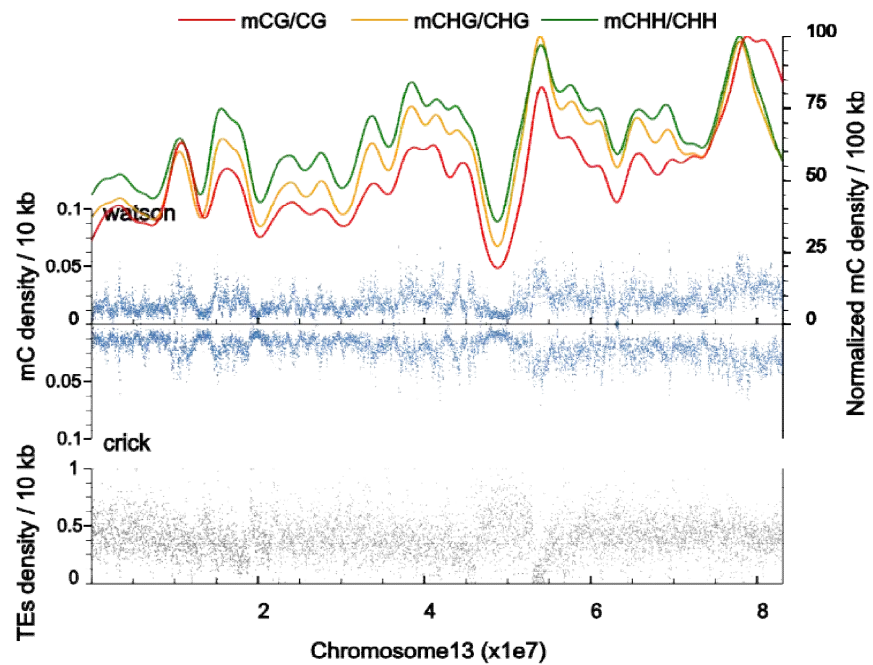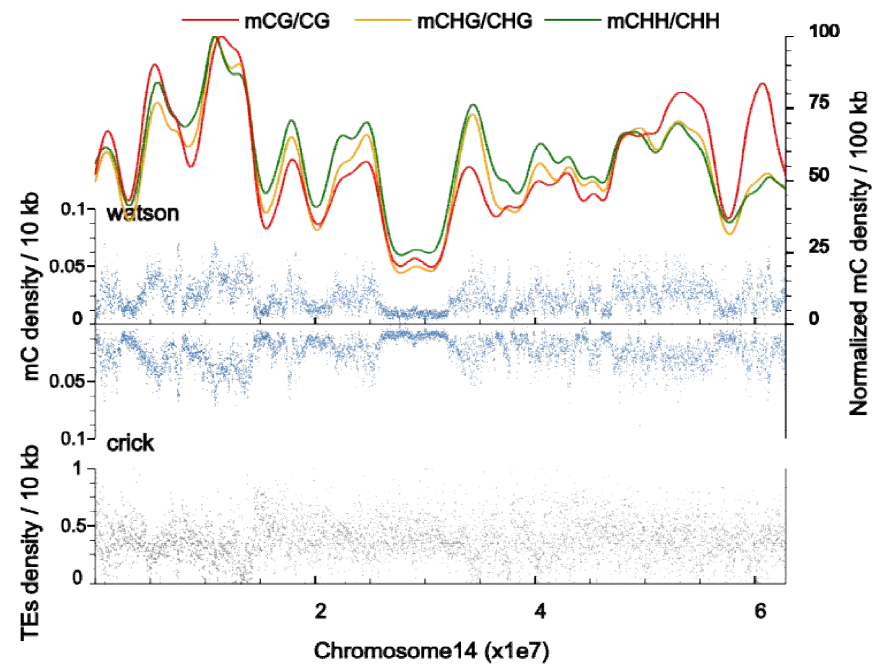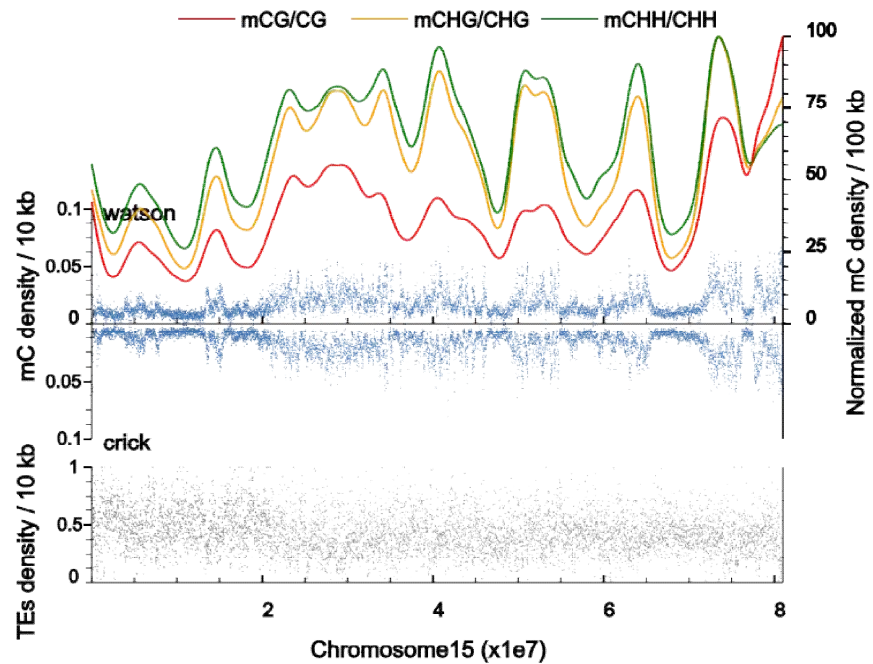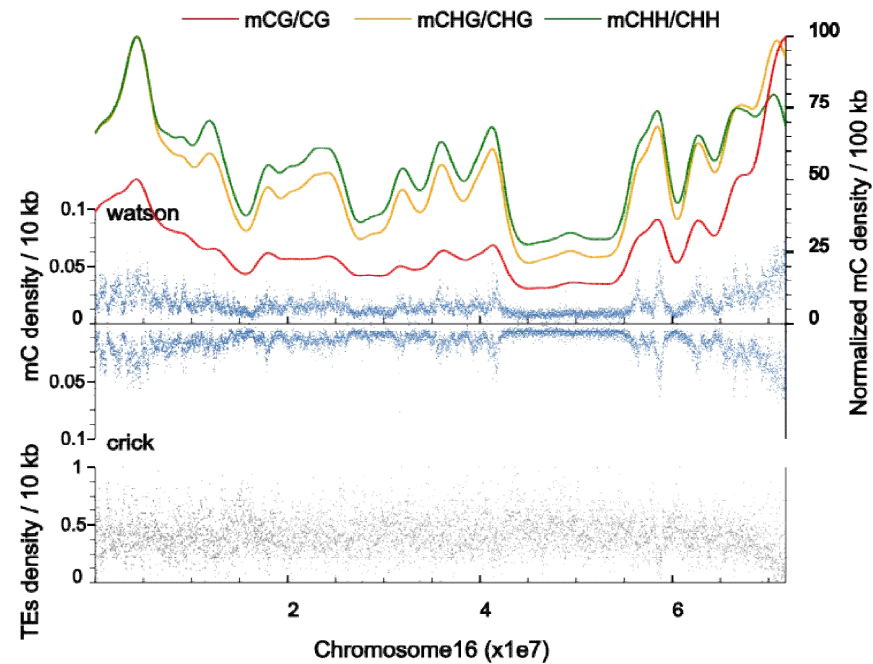

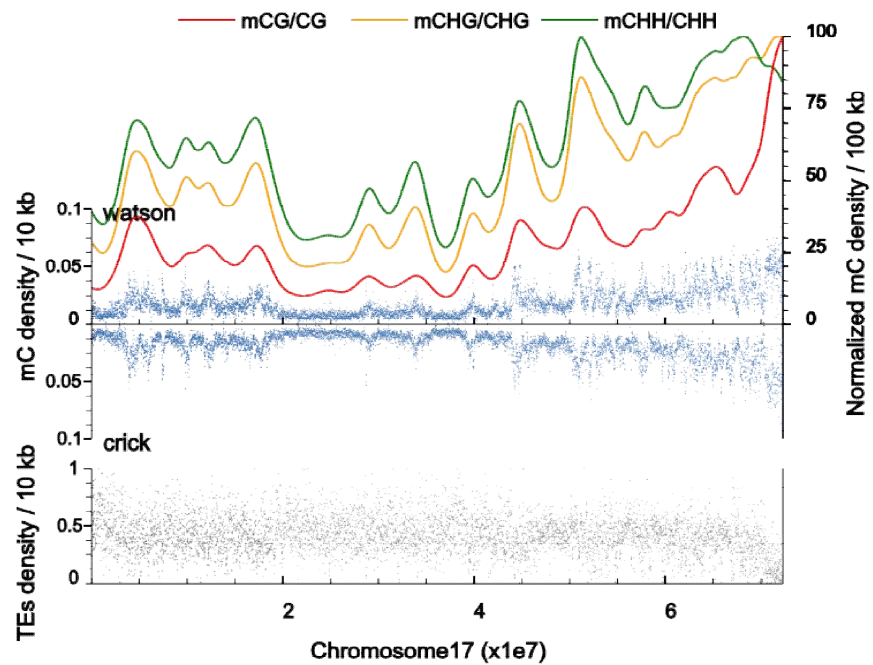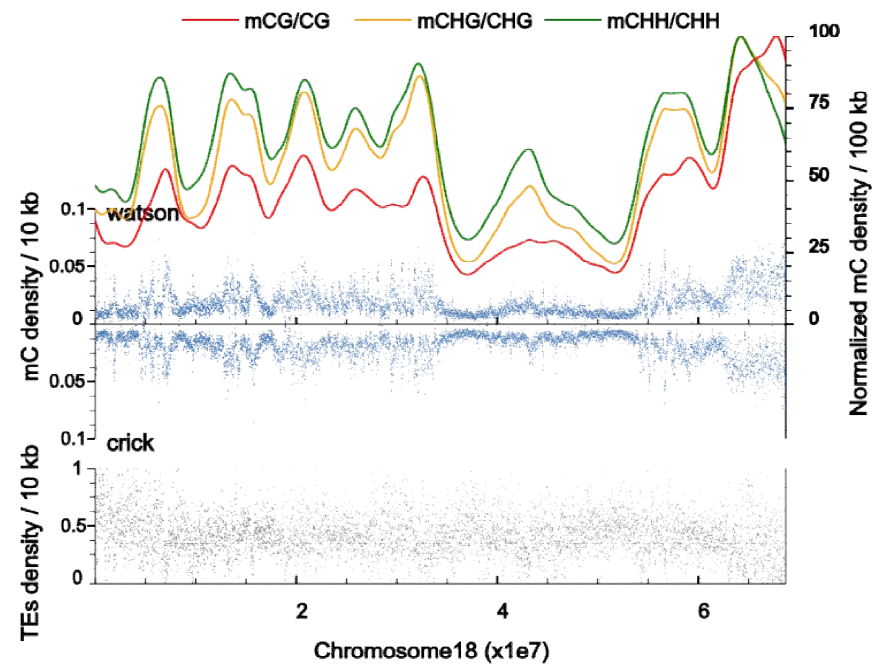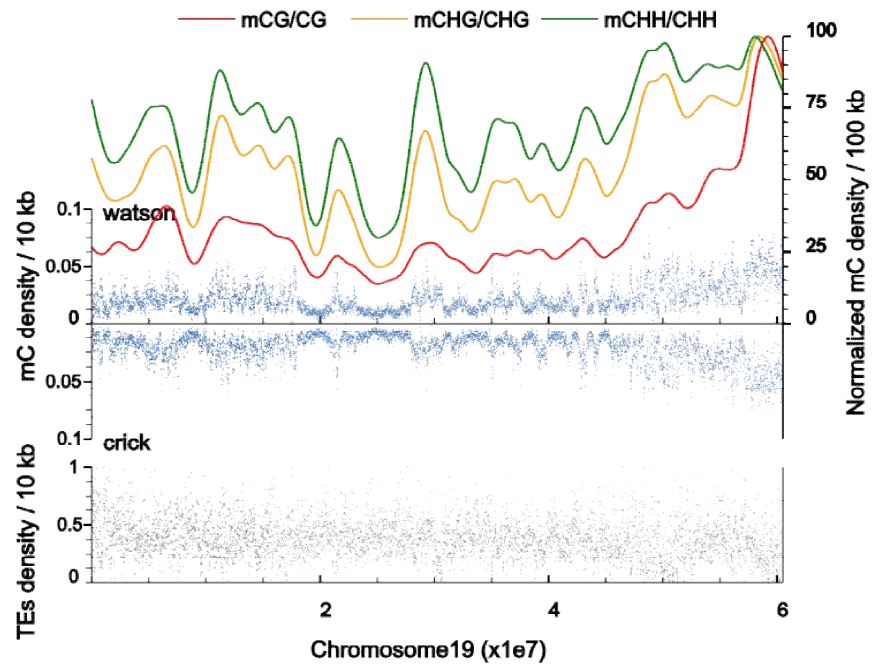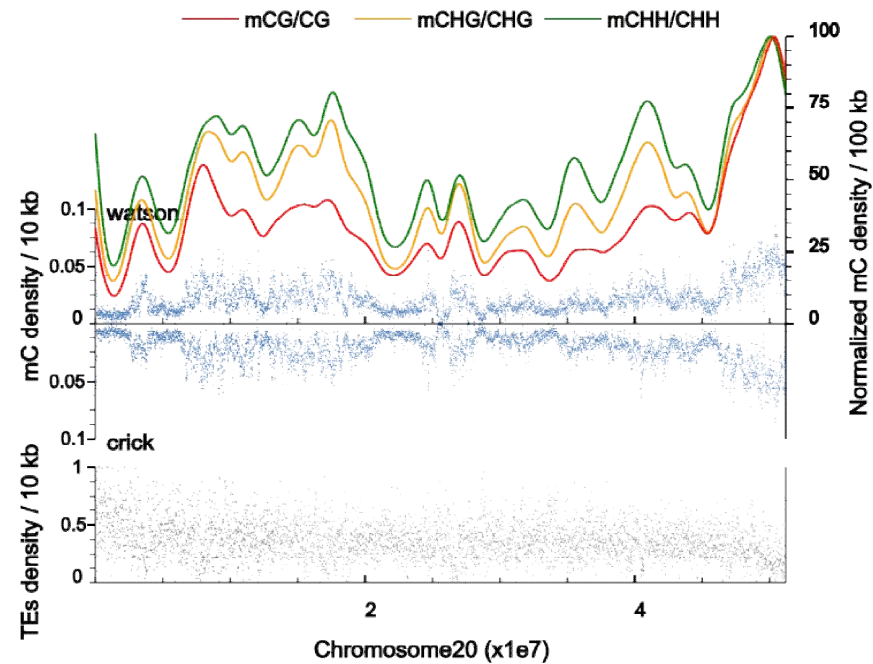

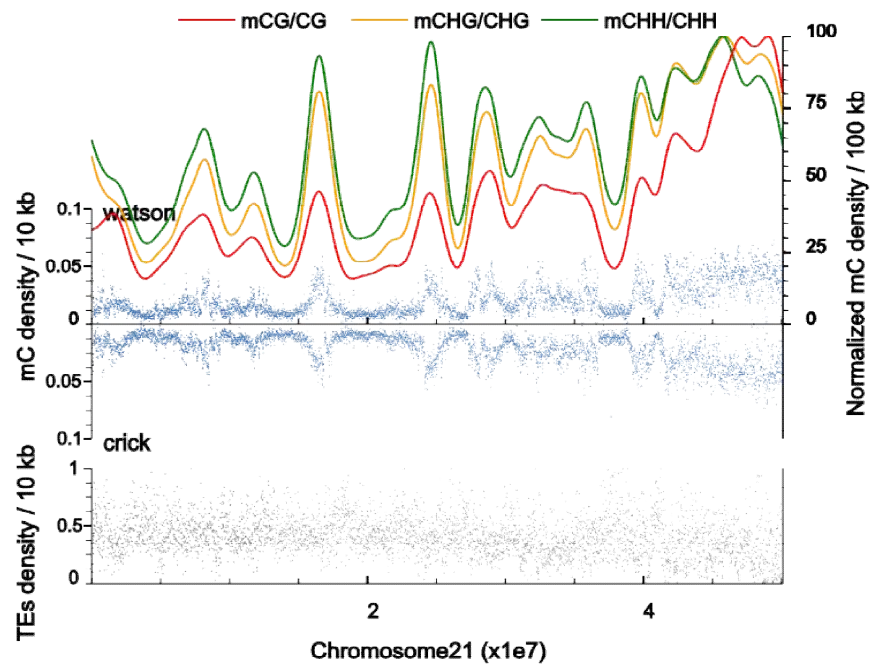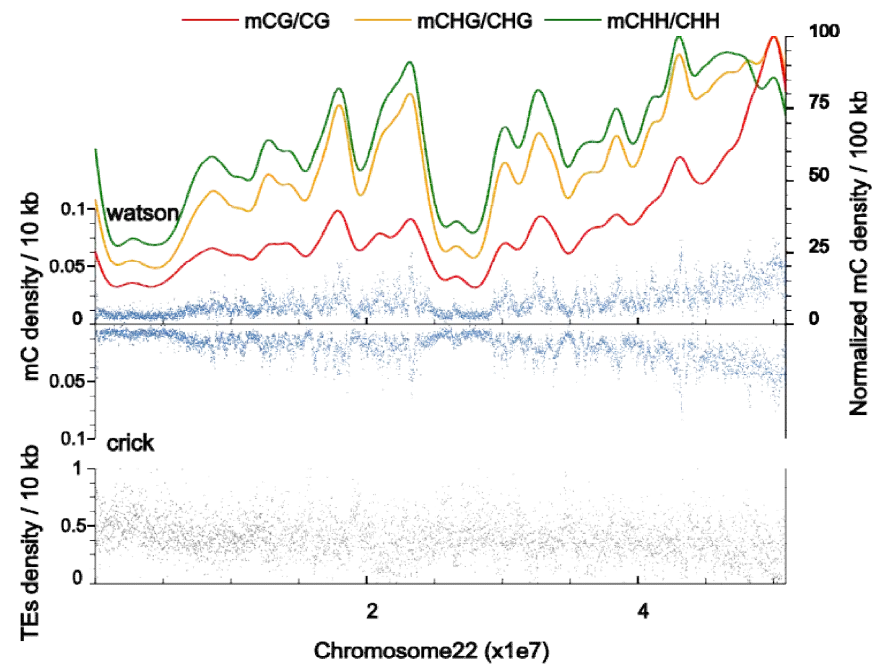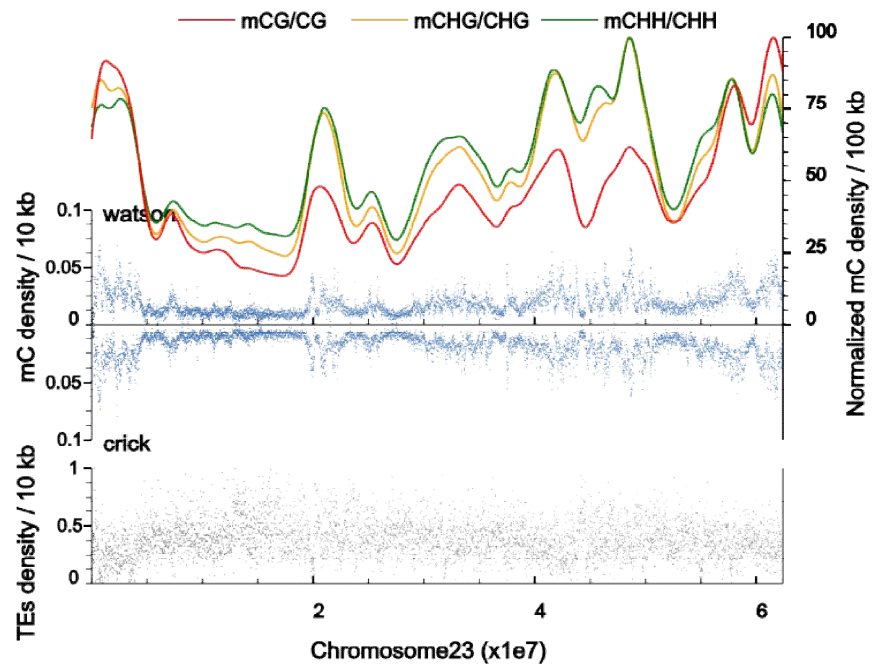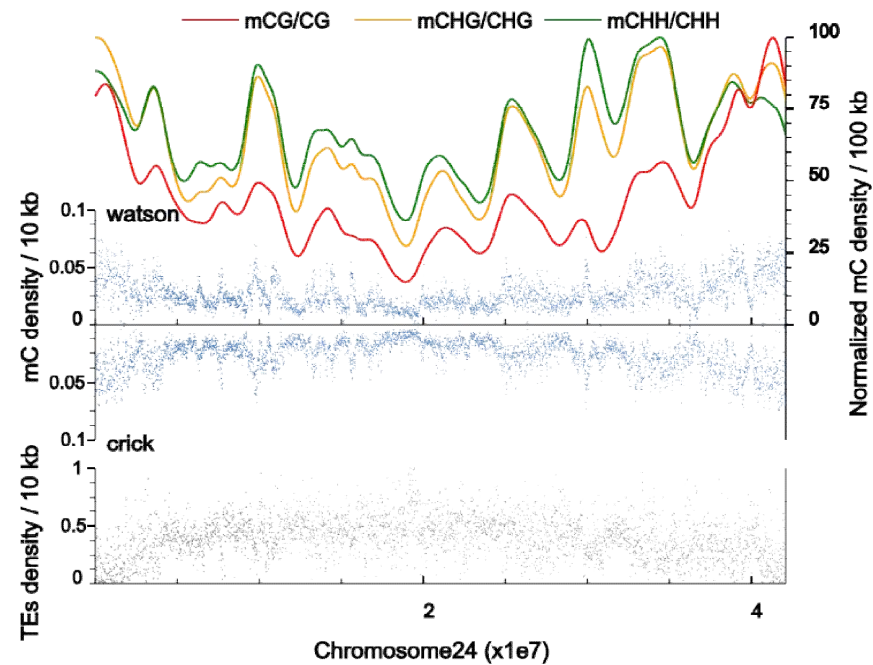

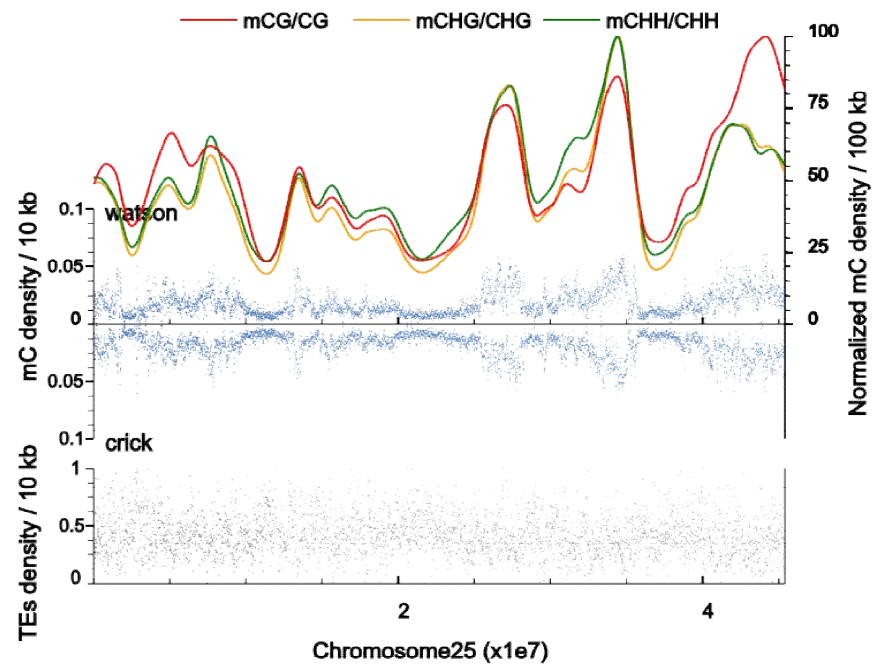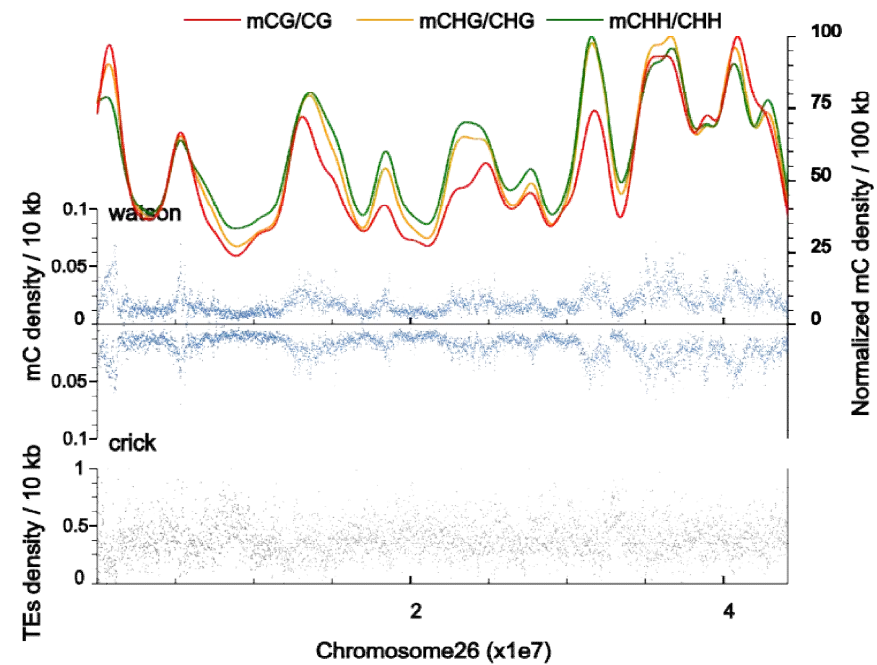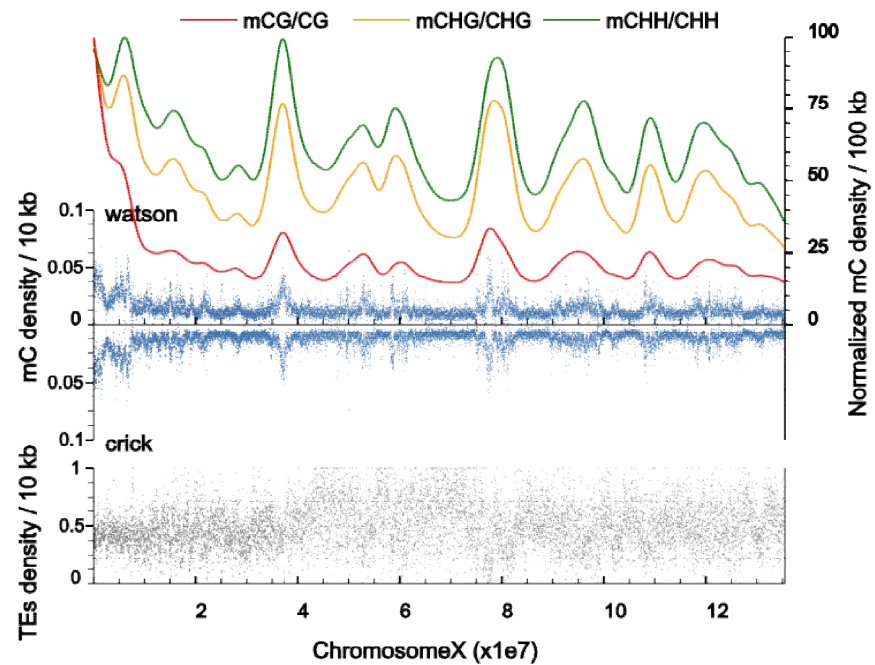

Supplement: S2 Fig — X-axis presents length of sheep chromosomes. Three contexts of mC density at 100 kb windows were profiled in normalized smoothed lines (red line stands for CG context methylation density, yellow line stands for CHG and green line stands for CHH). All mC density at 10 kb windows of both strands was profiled with blue scatter plots and repeats density at 10 kb windows was profiled with gray scatter plots. (PDF) [file pone.0142034.s002.pdf]
